# Examining lecture and inquiry-based laboratory performance for language minority students in science gateway courses

**DOI:** 10.1371/journal.pone.0267188

**Published:** 2022-04-28

**Authors:** Christian Fischer, Ha Nguyen, Gabriel Estrella, Penelope Collins

**Affiliations:** 1 University of Tübingen, Tübingen, Germany; 2 University of California, Irvine, CA, United States of America; University of Macau, MACAO

## Abstract

This study examined the effectiveness of lectures and inquiry-based instruction in supporting learning for language minority (LM) students in science gateway courses at a large public research university. Utilizing institutional data from 6,911 students across nine years, we fitted cross–lagged panel designs to model associations between lecture courses and inquiry-based laboratory courses for both LM and non-LM students in two-course sequences of introductory college Physics and Chemistry. We found that initial performance in lectures and laboratory sessions can be a predictor of subsequent course performance across disciplines and independent of LM status. Notably, while LM students performed worse in the initial lecture course, LM status resulted in neither worse performance in inquiry-based laboratory courses nor in worse performance in subsequent courses in the science gateway course sequence. Thus, this study suggests that interventions intended to support LM students in college science should target the initial courses in the corresponding science gateway course sequences.

## Introduction

As careers in science, technology, engineering, and mathematics (STEM) take greater precedence in the 21st-century job market, educational researchers and practitioners have begun to argue for greater integration of hands-on, authentic learning activities in science education. This is particularly true for the natural sciences, where inquiry-based learning through laboratory instruction has become crucial to successful STEM education. Inquiry-based instruction may be thought of as the educational practice whereby students construct knowledge by following the methods and practices used by professional scientists [1, 2]. When compared to traditional forms of lecture, inquiry-based learning environments have been found to significantly improve student learning, performance, and motivation in STEM [3–5]. However, despite these potential benefits, relatively few studies in higher education research have attempted to evaluate the effectiveness of inquiry-based laboratory instruction for language minority (LM) students [6–8]. As such, it remains unclear how inquiry-based instruction may support the academic achievement of LM students in postsecondary science courses [9].

Few would argue with the fact that science learning occurs not only within the lecture hall but also in laboratories in higher education settings. Though most current research focuses on modifying lecture-based instruction to be more aligned with more active learning and inquiry-based models of learning [5, 10], laboratory courses are particularly well suited for supporting inquiry-based learning environments [11–13]. Interestingly, previous studies indicated that student participation in laboratory experiments had positive effects on student learning and academic achievement [14, 15]. Indeed, the experiments conducted by students in laboratory courses are intended to foster the self-directed learning processes that characterize inquiry-based instruction [2]. Therefore, we intend to better understand the importance of laboratories as an inquiry-based environment to student learning in postsecondary science education. *The primary objective of this study is to investigate associations of inquiry-based instruction and how they compare to traditional lecture-based instruction on student achievement in subsequent college science courses with a focus on LM students*.

## Theoretical framework

There are compelling reasons for choosing inquiry over more traditional teaching methods in higher education settings. Inquiry instruction is thought to produce more meaningful learning outcomes than direct instruction due to the deep levels of processing that occurs when students attempt to make sense of, explain, and communicate their scientific findings with others [16]. This advantage is often mirrored with findings that information is better remembered when it is actively self-generated (e.g., explaining the process of photosynthesis to oneself or a fellow peer) rather than passively consumed (e.g., solely reading about photosynthesis in a textbook) [17]. To this degree, recent textbooks even incorporate active learning elements though direct links to computer-based simulations alongside experimentation and reflection tasks (for instance, “Wave Motion as Inquiry” for introductory college level Physics) [18].

Indeed, active learning has been found to increase undergraduate student achievement in STEM classes by almost half a standard deviation when compared to traditional lectures [5]. Similarly, a recent study found that students in courses taught with textbooks focused on inquiry learning showed better performance compared to courses using more traditional textbooks [19]. Inquiry-based instruction is also believed to be exceptionally well-suited for building students’ problem solving and critical thinking skills, as it requires students to construct knowledge for themselves by engaging in sophisticated forms of scientific reasoning in the context of authentic problem solving, as opposed to merely relying on rote memorization [20]. In addition to these academic and cognitive benefits, research on academic motivation suggests that the hands-on, collaborative nature of inquiry instruction increases students’ positive attitudes and intrinsic motivation towards learning science [21–24].

Because of its cognitive and motivational benefits, inquiry-based instruction may be particularly beneficial for students in need of greater support. A long-standing body of research in postsecondary settings provides strong evidence of persistent disparities in retention and graduation rates for students from underrepresented minority groups, including language-minority students from non-English speaking households and those who are continuing-generation, nonminority students [25, 26]. Numerous studies show an achievement gap for LM students in STEM courses and that they are more likely to leave STEM majors [6, 27–29]. On the one hand, the greater attrition rate for LM students may reflect the perceived chilly climate of STEM, or the student perception that faculty, and other instructors are unapproachable, intimidating, cold, and indifferent [30, 31]. On the other hand, given the growing proficiency in English for LM students, the achievement-gap may reflect the complexity of scientific language [32], such as its informational density, heavy use of infrequent words, and technical vocabulary [33, 34]. Inquiry-based instruction, with its emphasis on experimentation and student-constructed knowledge, may mitigate the language-demands of science and decrease the achievement gap for LM students. Further, inquiry-based instruction may provide a more inclusive learning environment for LM students, as it fosters an environment of collaboration and group work, encourages discussion among students and increases the perceived availability and approachability of instructors [35]. A recent meta-analysis reported that active learning in STEM courses reduced the achievement gap for minority and low-income by 33% in their examination grades and reduced the gap in pass rates by 45% [36]. Further, moderator analyses revealed increasing the intensity or class-time devoted to active-learning activities yielded narrower achievement gaps for minoritized groups of students. Similarly, studies that examined mediation effects found that increases in student self-efficacy mediated the increase in student performance due to active learning pedagogy in particular for URM students [37]. Despite these promising findings, it is noteworthy that laboratory-based classes were just one of several types of active learning activities examined in the aforementioned meta-analysis, including activities such as problem-based learning, collaborative groups, and the use of clickers. Because the goal was not to examine each type of activity’s pedagogical benefits, it is unclear the degree to which inquiry-based laboratories may affect the achievement gap for URM students. That said, a recent study that examined the pairing of lecture courses with accompanying laboratory sessions did not find significant effects on student performance by different subpopulations of students, while overall indicating that laboratory participation increased students’ lecture performance [14].

While many studies find that active learning may enhance equitable STEM learning opportunities for minoritized groups of students [24, 36–38], it is less clear whether it would have differential benefits for LM students. Although much of the higher education literature has addressed the underrepresentation of women, low-SES and black, indigenous, and other people of color (BIPOC) in STEM, relatively little higher education research has focused on serving LM students [29, 39]. Further, the LM student population is heterogeneous, varying widely in the proficiency in English, academic preparedness, and SES. Although LM students in general are more likely to enroll in community colleges than their non-LM peers and less likely to enroll in non-selective four-year institutions. However, at selective four-year institutions the proportion of LM students is comparable to the proportion of non-LM students [7]. This bifurcation may reflect important differences in the LM populations, as LM students attending selective four-year institutions may be more English-proficient than those who attend community colleges and less selective institutions. Despite their greater English-proficiency, little is known about how best to serve LM students at selective four-year institutions. To this end, we ask the following research questions (RQs):

*RQ1*: What differences in academic preparedness, language ability, and demographic background exist between LM and non-LM students?
*RQ2*: How are initial student performance in inquiry-based labs and lectures associated with subsequent course performance in inquiry-based labs and lectures?
*RQ3*: How are associations of initial student performance in inquiry-based labs and lectures with subsequent performance in inquiry-based labs and lectures different between LM and non-LM students?

## Methodology

### Data sources and study setting

The current study is situated at a large public research university in California. The university has been federally designated as a Hispanic-Serving Institution (HSI) and an Asian American and Native American Pacific Islander-Serving Institution (AANAPISI). Data for this study has been provided from multiple institutions on campus including the Registrar’s Office, the Office of Institutional Research, the Office of Information Technology, and Admission, among others. This study utilizes seven cohorts of students (2009–2017) who enrolled in two large introductory science gateway course series (i.e., General Chemistry and Classical Physics). This study was approved by the UC Irvine Institutional Review Board (HS#2018–4801). Informed consent was not required.

Each course series required students to simultaneously enroll in a lecture course with an associated inquiry-based laboratory session in the same term for two subsequent terms. The laboratory experiments were closely tied to the corresponding lecture content knowledge in each term. Notably, these two-course series represent the only such science course series at this university. For Chemistry, this two-term course series (with the unique lecture-laboratory pairing in the same term) was only offered in 2009–2011, whereas it was offered in 2009–2017 for Physics.

Topics of the Chemistry course series included properties of gases, liquids, solids, and solutions; changes of states; and energetics of chemical reactivity (course 1); as well as equilibria, aqueous acid-base equilibria, solubility equilibria, electrochemistry, and nuclear reactions (course 2). Topics of the Physics course series included force, energy, momentum, rotation, and gravity (course 1) and electricity and magnetism (course 2). Our study only investigates students who enrolled in all four courses of the corresponding course series in the same academic year. We excluded international students as their language experiences may differ from U.S. born students, which may confound results of the analysis. Also, we excluded transfer students as these courses are introductory gateway courses that are typically taken early in students’ college career. Transfer students who enroll in these courses may have already enrolled in similar courses, which may introduce underlying biases. Robustness checks that included both international and transfer students indicated that the results remained similar to the more conservative analytical sample of the study (see Tables A1, A2 and Figs A1-A4 in S1 File).

The analytical sample of this study includes 6,911 students (2,865 Chemistry students; 4,046 Physics students). About 61% of students enrolled in the Chemistry course series are female (39% male), whereas 26% of students in the Physics course series are female (74% male). Most students have an Asian, Asian American, or Pacific Islander racial/ethnic background (60% Chemistry, 51% Physics), followed by White (15% Chemistry, 20% Physics), and Latino and Hispanic (13% Chemistry, 22% Physics). Notably, 19% of students are language minority students in the Chemistry course series, whereas 25% of students are language minority students in the Physics course series. Table 1 provides full demographic information of the study sample.

**Table 1:** Demographic information of study samples by language group.

	Combined		LM students		Non-LM students	
	N	%	N	%	N	%
Physics						
Home language						
English only	1,586	39			1,586	52
English and another language	1,436	35			1,436	48
Another language	1,024	25	1,024	100		
Race/Ethnicity						
White	792	20	74	1	718	25
Black/African American	66	2	5	0	61	2
Latino/Hispanic	851	22	324	32	527	18
Asian/Asian American/Pacific Islander	1,984	51	548	54	1,436	49
Other	235	6	57	6	178	6
Gender						
Male	2,976	74	719	70	2,257	75
Female	1,056	26	304	30	752	25
Chemistry						
Home language						
English only	961	34			961	41
English and another language	1,369	48			1,369	59
Another language	535	19	535	100		
Race/Ethnicity						
White	421	15	54	10	367	16
Black/African American	72	3	8	2	64	3
Latino/Hispanic	365	13	102	20	263	12
Asian/Asian American/Pacific Islander	1,662	60	321	62	1,341	60
Other	233	8	33	6	200	9
Gender						
Male	1,127	39	189	35	938	40
Female	1,729	61	346	65	1,383	60

*Notes*. LM: Language minority; percentages may not add up to 100% due to rounding.

### Measures

The *dependent variable* is a continuous variable indicating student course performance on a 4.0 scale (A+ = 4.0, A = 4.0, A- = 3.7, B+ = 3.3, …, D = 1.0, D- = 0.7, F = 0). The letter to numerical grade conversion mirrors the university grading policy.

The core *independent variable* of interest represents whether students come from a language minority background. The categorization is based on student self-report data of growing up in monolingual-English, bilingual-English, or non-English homes. As academic and background differences between monolingual- and bilingual English students proved negligible, these categories were collapsed to a non-LM comparison group. To adjust for potential confounding with underlying differences in students’ demographic and academic backgrounds [5, 7, 40], two blocks of *covariates* were included in analyses, academic preparedness and family background. Academic preparedness controls included the number of pre-college enrollment units, as well as students’ mathematics, reading, and writing scores of college admission tests (i.e., ACT/SAT examinations). Family background characteristics included family household size, first-generation college student status (i.e., neither parent received a bachelor’s degree), low-income status (as classified based on household income and size at the 185% U.S. poverty line cutoff), and fathers’ education level. Notably, fathers’ educational level was used as model fit indices were better compared with mothers’ educational level (see Table A3 in S1 File). That said, robustness checks indicated that the findings are similar in both magnitude and direction (see Fig A5 in S1 File).

Missing data on the LM-variable was list-wise deleted as it represents the grouping variable. For all other variables, the analyses applied full information maximum likelihood (FIML) approaches [41].

### Analytical methods

*Research question 1* uses descriptive analysis on students’ academic preparedness, language ability, and socio-demographic background variables and estimated standardized mean differences between LM and non-LM students, as well as the corresponding effect sizes (Cohen’s *d*).

*Research question 2* utilizes structural equation modeling to examine whether inquiry-based lab instruction was associated with greater student performance compared to traditional lecture-based instruction. A cross-lagged panel design modeled the reciprocal effects of both inquiry and lecture-based instruction on subsequent course performance [42]. Latent constructs measuring students’ academic preparedness, language ability, and family background were included as covariates to control for the potentially confounding influences [43]. Model parameters used maximum likelihood estimation with Satorra-Bentler scaled chi-squared values and robust standard errors to adjust for biases associated with non-normal data. All models exhibited acceptable model fit; CFI > .90, TLI > .90, RMSEA < .08, SRMR < .08 [44].

*Research question 3* fits multi-group structural equation models to examine whether associations of lecture and inquiry-based lab instruction with subsequent course achievement differed by LM status. This analysis used χ^2^ difference test to provide evidence of moderation [45], comparing the model fit of constrained single-sample models (path estimates fixed to equality based on LM status) to nested multi-group models (path estimates freed to vary between groups).

## Results

### Academic preparedness and demographics by language background (RQ1)

To compare students’ academic preparedness and family background characteristics, we calculated the effect sizes of the standardized mean differences by LM status (Table 2). Regarding students’ academic preparedness, LM students entered the university with substantially lower reading scores (Chemistry: *d* = .31, *p* < .001; Physics: *d* = .32, *p* < .001) on the college admission exam for both course series when compared to non-LM students. Similarly, LM students’ writing scores on the college admission exam were significantly lower for both course series (Chemistry: *d* = .19, *p* < .001; Physics: *d* = .18, *p* < .001), when compared to non-LM students, although the effect sizes are considerably smaller. In Physics, LM students had significantly lower mathematics scores on the college admission exam (*d* = .15, *p* < .01) compared to non-LM students, whereas in Chemistry students did not significantly differ on their mathematics scores by LM status (*d* = 0.04, *p* = .94). Interestingly, LM students entered the college with more pre-college enrollment units in Physics (*d* = .13, *p* < .001), whereas this difference was negligible for Chemistry (*d* = .03, *p* = .06).

**Table 2:** Descriptive statistics and standardized mean differences by language group.

	LM		Non-LM		Effect size and 95% CI		
	M	SD	M	SD	d	Low CI	High CI
Physics							
Course grade							
Course 1 Lec	2.80	.85	2.86	.80	.08*	.01	.15
Course 1 Lab	3.78	.58	3.78	.56	-.00	-.07	.07
Course 2 Lec	2.76	.97	2.74	.96	-.02	-.09	.05
Course 2 Lab	2.98	.68	2.98	.61	.01	-.07	.08
Academic preparedness							
Entry units	24.12	19.89	26.70	19.47	.13**	.06	.20
Math score	83.01	9.92	84.44	9.41	.15**	.08	.22
Reading score	71.78	13.20	75.91	12.61	.32***	.25	.39
Writing score	72.84	12.03	75.04	12.00	.18***	.11	.25
Family background							
Father’s education	3.70	2.07	5.08	1.80	.74***	.66	.81
Low-income status	54%		23%				
First-generation status	49%		47%				
Household size	4.29	1.28	4.16	1.14	-.11**	-.19	-.03
Chemistry							
Course grade							
Course 1 Lec	2.45	1.02	2.61	.92	.18***	.09	.27
Course 1 Lab	3.23	.54	3.23	.58	.02	-.09	.13
Course 2 Lec	2.33	1.07	2.37	1.04	.03	-.08	.15
Course 2 Lab	3.08	.69	3.03	.68	-.07	-.18	.05
Academic preparedness							
Entry units	23.69	18.54	24.27	19.56	.03	-.06	.12
Math score	74.16	12.61	74.66	11.37	.04	-.05	.14
Reading score	63.51	14.24	67.51	12.75	.31***	.21	.40
Writing score	66.06	13.57	68.37	11.85	.19***	.09	.28
Family background							
Father’s education	4.29	2.08	5.12	1.80	.45***	.35	.55
Low-income status	44%		23%				
First-generation status	47%		32%				
Household size	4.23	1.07	4.30	1.19	.06	-.04	.16

*Notes*. LM: Language minority; lower and upper bounds of the 95% Confidence Interval (CI); positive value indicate direction of effect in favor of non-LM students, whereas negative values are in favor of LM students

*p < .05

**p < .01

***p < .001.

Regarding student family characteristics, LM students came from less-formally educated households (Chemistry: *d* = .45, *p* < .001; Physics: *d* = .74, *p* < .001). Household sizes did not substantially vary by LM-status. Also, more LM students were classified as low-income students compared to non-LM students (Chemistry: 44% LM vs. 23% non-LM; Physics: 54% LM vs. 23% non-LM). Similarly, more LM students were first-generation college students in chemistry (47% LM vs. 32% non-LM), although the percentage is similar for Physics (49% LM vs. 47% non-LM). Overall, these findings suggest that LM students may enter the university relatively less prepared to perform in introductory science gateway courses than non-LM students.

### Subsequent student performance in inquiry-based labs and lectures (RQ2)

**Fig 1:**
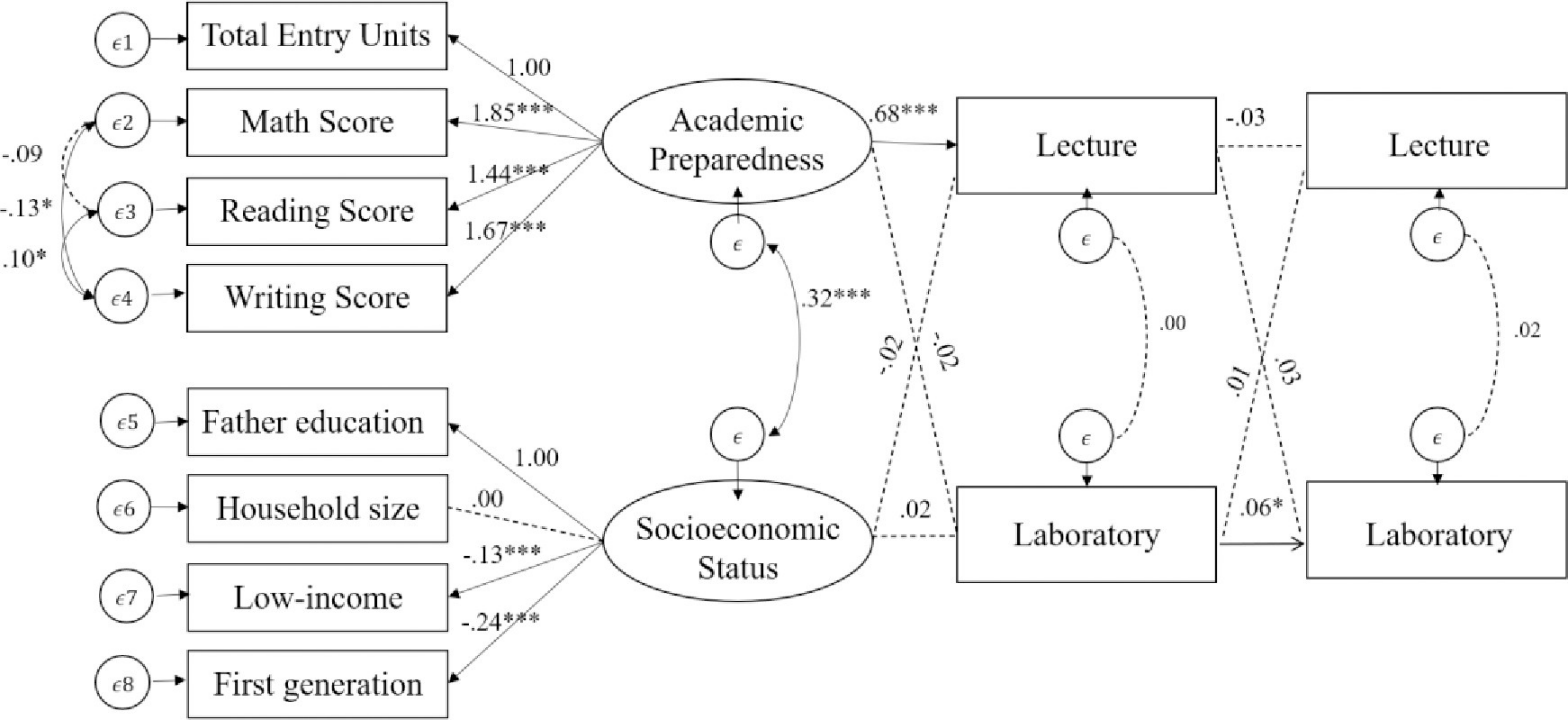
Cross-lagged panel analysis of chemistry lecture and inquiry-based lab course sections. CFI = .97, TLI = .96, RMSEA = .04 [.03, .04], SRMR = .03, N = 2,865; p < .05., **p < .01., ***p < .001.

Cross-lagged panel models examined whether inquiry-based labs were stronger predictors of student achievement in subsequent courses than lecture-based instruction. For Chemistry (Fig 1), initial laboratory performance was a statistically significant predictor of subsequent laboratory achievement, *β* = .06, *p* <. 05, but not of subsequent lecture performance, *β* = .01, *p* > .05. Initial lecture performance did neither significantly predict subsequent lecture performance, *β* = -.03, *p* > .05., nor subsequent laboratory performance, *β* = .03, *p* > .05. Interestingly, students’ academic preparedness was only significantly predictive of students’ initial lecture performance, *β* =. 68, *p* < .001, but not for students’ initial laboratory performance, *β* = -.02, *p* > .05.

**Fig 2:**
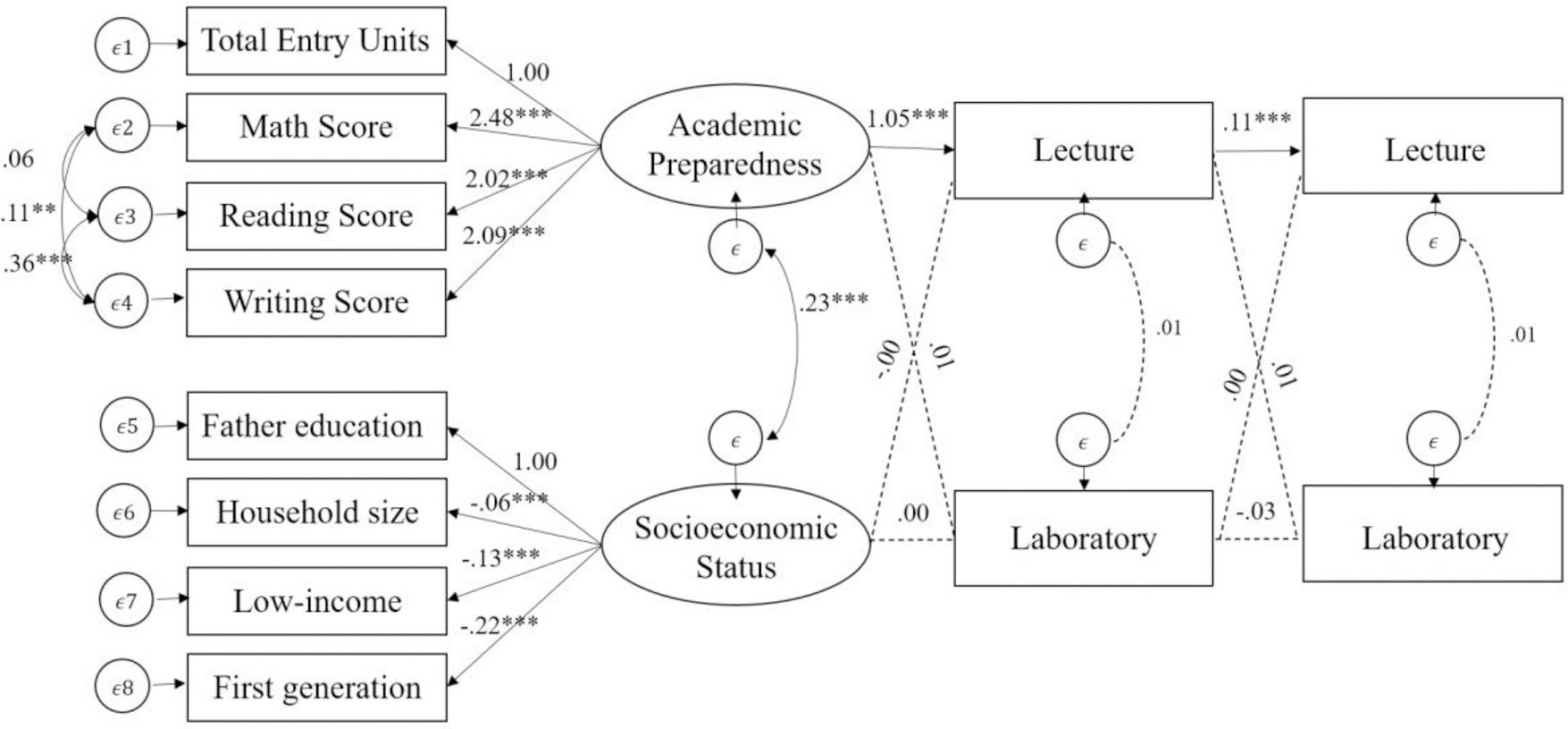
Cross-lagged panel analysis of physics lecture and inquiry-based lab course sections including transfer and international students. CFI = .99, TLI = .99, RMSEA = .02 [.02, .03], SRMR = .02; N = 4,046; p < .05., **p < .01., ***p < .001.

For Physics (Fig 2), students’ academic preparedness was similarly only significantly predictive of students’ initial lecture performance, *β* = 1.05, *p* < .001, but not for students’ initial laboratory performance, *β* = .01, *p* > .05. In contrast to Chemistry, students’ initial lecture performance was a statistically significant predictor of subsequent lecture performance, *β* = .11, *p* <. 05, but it did not significantly predict subsequent laboratory performance, *β* = .01, *p* > .05. Similarly, initial inquiry-based lab performance did neither significantly predict subsequent lecture performance, *β* = .00, *p* > .05., nor subsequent laboratory performance, *β* = -.03, *p* > .05.

### Achievement in inquiry-based labs and lectures across language groups (RQ3)

**Fig 3:**
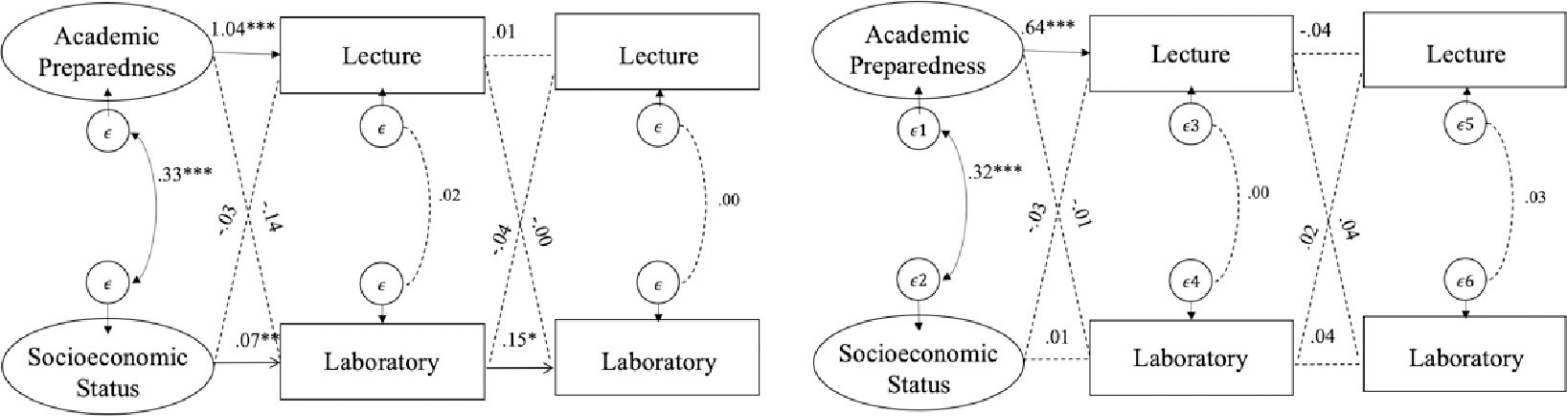
Multi-group structural equation model representing the associations between lecture and inquiry-based lab performance in chemistry course series. left: LM students, right: non-LM students; latent covariates for academic preparedness and SES are included but not shown; dashed lines describe non-significant path estimate; *p < .05., **p < .01., ***p < .001.

Multi-group structural equation models examined whether the impact of lecture and inquiry-based lab instruction on subsequent course achievement differed between LM and non-LM students. For chemistry (Fig 3), only LM-students had a significant association between initial and subsequent performance, namely the initial laboratory performance on subsequent laboratory performance (*β* = .15, *p* < .001). However, a chi-square test of equality indicated that this association did not differ between language groups (*χ*^*2*^ = 2.63, *p* = .10). Interestingly, academic preparedness had a significantly higher influence on initial lecture performance for LM students (*β* = 1.04, *p* < .001) compared to non-LM students (*β* = .64, *p* < .001), *χ*^*2*^ = 6.33, *p* < 0.05. All other paths were not significantly different across LM groups.

**Fig 4:**
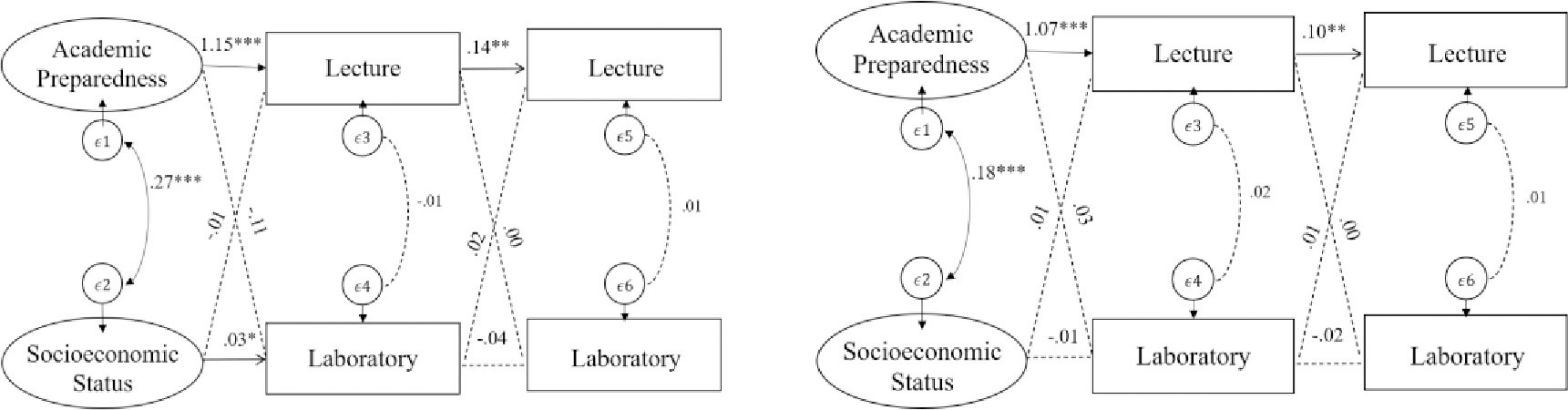
Multi-group structural equation model representing the associations between lecture and inquiry-based lab performance in physics course series. left: LM students, right: non-LM students; latent covariates for academic preparedness and SES are included but not shown; dashed lines describe non-significant path estimate; *p < .05., **p < .01., ***p < .001.

For physics (Fig 4), initial lecture-performance was a statistically significant predictor of subsequent lecture achievement for both LM students (β = .14, p < 0.01) and non-LM students (*β* = .10, *p* < .01) students. However, a chi-square test of equality indicated that this association did not differ between language groups (*χ*^*2*^ = 169.53, *p* = .27). Also, social economic status had significantly higher influence on students’ initial lab performance for LM students (*β* = .03, *p* > .05) compared to non-LM students (*β* = -.01, p < .05), *χ*^*2*^ = 7.75, *p* < 0.05. All other paths were not significantly different across LM groups.

## Discussion

This large quantitative study extends the research base two-fold. This study contributes to discussions on the antecedents and benefits of inquiry-based and traditional lecture-based instruction for student learning in higher education. Also, this study extends these discussions to students with linguistically diverse backgrounds, which are currently underrepresented in the literature. The two main findings of this study are as follows:

First, this study suggests that initial performance in lectures or laboratory sessions can be a predictor of subsequent course performance in corresponding lecture and laboratory courses, depending on the disciplinary content area. These findings support the somewhat divergent literature base. One the one hand, researchers argue that lectures can be more effective mode of delivering complex information without detriment to student learning and performance [9, 46, 47]. This correspond to our findings regarding the Physics course series indicating that initial lecture performance was only associated with subsequent lecture performance but not with subsequent laboratory performance (while initial Physics laboratory performance predicted neither subsequent lecture or laboratory performance). On the other hand, other studies argue that inquiry-based learning is more effective than traditional lecture-based instruction [5, 10, 48]. This corresponds to our findings for the Chemistry course series indicating that initial laboratory performance predicted subsequent laboratory performance (but not subsequent lecture performance). This suggests that both disciplinary content and institutional context matter. Therefore, college instructors and educational policy-makers are encouraged to carefully consider their particular disciplinary content area when suggesting subsequent benefits of inquiry-based or lecture-based instruction.

Second, although LM students entered college with less beneficial academic preparation, compared to non-LM students–which mirrors current literature [49]–we did not find support that LM status influenced how initial lecture and inquiry-based instruction impacted subsequent performance in the course series. That said, we still found that LM students had overall lower courses grades in the initial lecture course but not the initial inquiry-based laboratory course, which aligns with research that suggests that active learning may be a means of reducing the achievement disparities often experience by LM and URM students [16, 26, 35, 50]. Further, our study indicates that LM students are not additionally penalized in subsequent lecture or laboratory courses in science gateway course series.

These findings have important implications for STEM education. Given that academic preparedness, rather than LM status, contributed to student performance in the initial course, we encourage educational administrators and university instructors to aim interventions and support systems at the first course in college gateway science courses sequences for LM students and others who may be less well prepared, such as first generation or low SES students. These may include short preparatory online courses with foci in the intersection of the science content and the challenges posed by scientific language prior to the first course [51, 52] or nudging intervention to increase motivation, self-efficacy and self-regulatory abilities [37, 53, 54].

### Future work

With the increased availability of large institutional data sets at many universities [55], we encourage researchers to replicate this study at their own institutions to mirror the nationwide diversity in student demographics and institutional contexts to assess the generalizability of our findings. We particularly encourage these replications to take place at community colleges, as most LM students attend community colleges rather than four-year institutions [7, 56]. There are important differences between LM students who attend selective four-year universities, such as those in our study, and LM students attending less selective institutions and community colleges, particularly in terms of their English proficiency and academic preparedness [7]. Also, we encourage researchers to utilize additional data sources that are available at scale to supplement such analysis. Potential data sources may include clickstream data from the learning management systems that capture student behavior during the science courses [57]; or data from college entrance surveys, which may include measures of stable psychological constructs such as student personalities characteristics [58]. From a methodological perspective, this study utilizes a correlational research design which is common for such institutional research. That said, future research may attempt to get closer to generate causal inference, for instance through a randomized controlled field design. However, the university setting may posit some practical limitation hindering random sampling, for instance, regarding student course selection. In addition, future work may uncover factors that contribute to the performance gap of LM students in initial lecture classes. Such factors may include student-level factors such as self-regulatory skills, student motivation, and study skills [59–61]; instructor-level factors such as teaching quality, teaching experience, instructional styles, and choice of the textbook [5, 19, 62]; and context-level factors such as scheduling of the classes, department funding, and the perceived chilly climate often reported in STEM learning environments [27, 63, 64]. Another strand of research may develop and evaluate interventions attempting to narrow this initial gap in lecture course performance for LM students such as short preparatory courses [51, 52]; or nudging interventions [53, 54].

## Conclusions

Our study adds to and extends the growing body of literature examining the more equitable approaches to STEM education for LM students [23, 28, 29]. Even among students enrolled at a selective research university, LM students, who were more likely to be first generation and come from lower SES backgrounds, had weaker academic preparation for higher education than their non-LM peers. For all students, weaker academic preparation contributed to worse performance in the initial lecture classes, which in turn contributed to weaker performance in subsequent lecture courses in the science gateway course sequences across all disciplines. Notably, while LM students performed worse in the initial lecture course, LM status resulted in neither worse performance in inquiry-based laboratory courses nor in worse performance in subsequent courses in the science gateway course sequence. The comparable performance of LM and their non-LM peers in the laboratory classes suggests that inquiry-based instruction and the active learning it promotes supports LM students and can be leveraged to enhance equity in STEM education. Further, this study suggests that colleges might adopt interventions to support LM students in college science that target the initial courses in the corresponding science gateway course sequences.

### Limitations

This study has a variety of limitations that should be considered when interpreting findings and implications. Most notably, data for this study was provided from students at a selective research university, which can be mirrored in the high academic preparedness scores (i.e., SAT/ACT performance) for both LM and non-LM students compared to a national average. Descriptive analyses for monolingual- and bilingual English students indicated a relatively small variance by LM status. Therefore, we decided to collapse these two student groups to a single non-LM students comparison group. This limits the generalizability of our findings, as well as the potential to detect finer-grained differences among LM students. That said, we would expect a larger magnitude in effect sizes in replication studies with more diverse student populations (e.g., community colleges, private universities, open enrollment universities). Additionally, we only examined two course sequences for two introductory science courses sequences (i.e., General Chemistry and Classical Physics) suggesting replication studies in other science disciplines (e.g., Biology) and for more advanced course series (e.g., organic chemistry) that were not available in these lecture-laboratory combinations at this university.

Methodologically, the models have an omitted variable bias as this study only included constructs that were already available within the institutional data sets. Other potentially important variables for explaining variance in student performance such as self-regulation, self-efficacy, interest in STEM, among others, as well as variables important for LM students (e.g., years of exposure to English, participation in scientific English courses or dual language programs) were not measured. Similarly, variables describing the instructional context in the analyzed courses beyond the lecture/laboratory classification were not measured at the institutional level and available for this study. Also, the study design is correlational in its nature; therefore, we cannot infer causal claims about the presented relationships.

## Supporting information

S1 File Additional information, data tables, and robustness checks. (DOCX)
