# Supplementary material for: Examining lecture and inquiry-based laboratory performance for language minority students in science gateway courses
Source: PLoS One. 2022 Apr 28;17(4):e0267188. doi: 10.1371/journal.pone.0267188 (PMC9049531; doi:10.1371/journal.pone.0267188)
Supplement: S1 File — (DOCX) [file pone.0267188.s001.docx]

**Supporting Information**


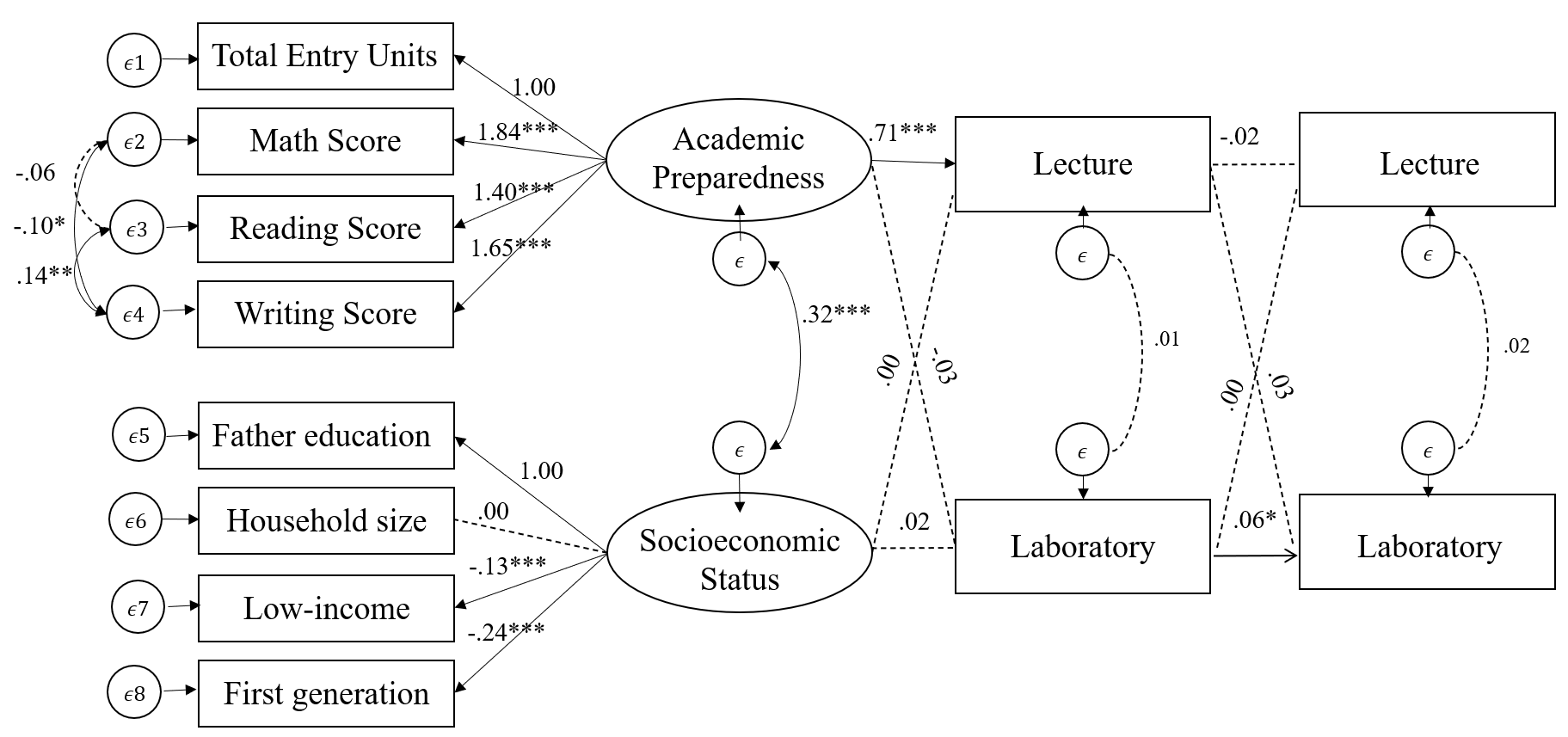


**Figure A1.** Cross-lagged panel analysis of chemistry lecture and inquiry-based lab course sections including transfer and international students; CFI = .97, TLI = .95, RMSEA = .04 [.03, .04], SRMR = .03, N = 2,911; p < .05., **p < .01., ***p < .001.


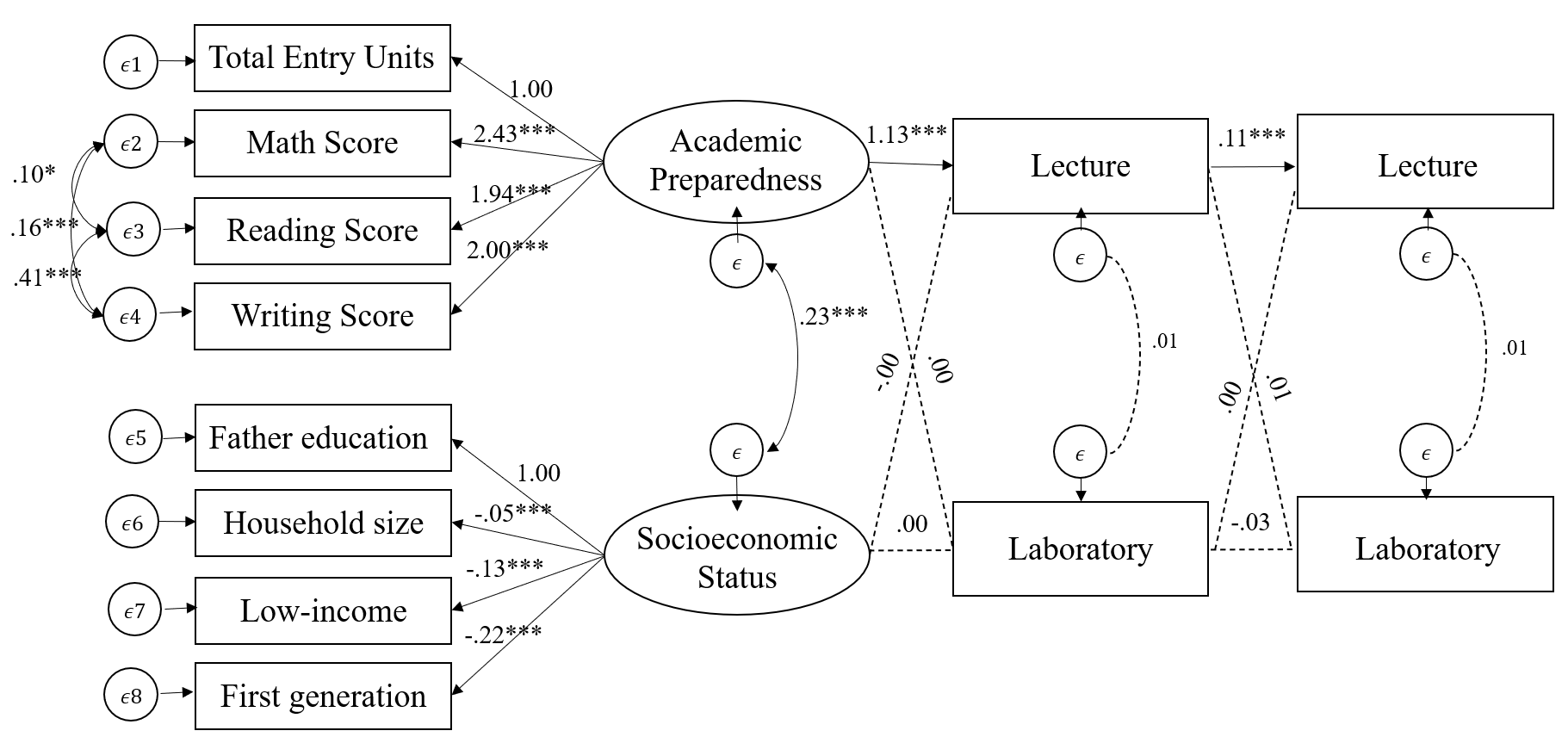


**Figure A2.** Cross-lagged panel analysis of physics lecture and inquiry-based lab course sections including transfer and international students; CFI = .99, TLI = .99, RMSEA = .02 [.02, .03], SRMR = .02, N = 4,741; p < .05., **p < .01., ***p < .001.


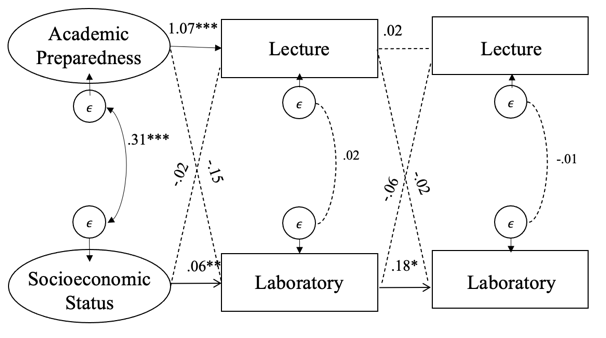

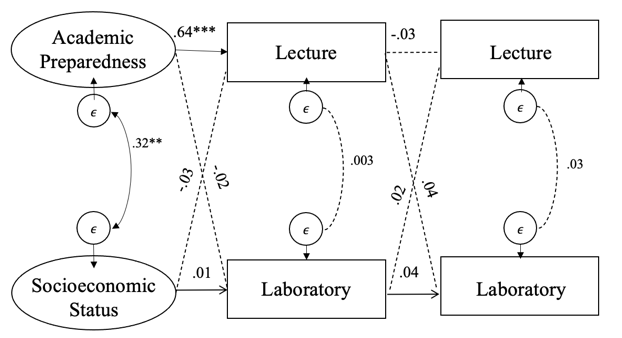


**Figure A3.** Multi-group structural equation model representing the associations between lecture and inquiry-based lab performance in chemistry course series including transfer and international students; left: LM students, right: non-LM students; latent covariates for academic preparedness and SES are included but not shown; dashed lines describe non-significant path estimate; *p < .05., **p < .01., ***p < .001.


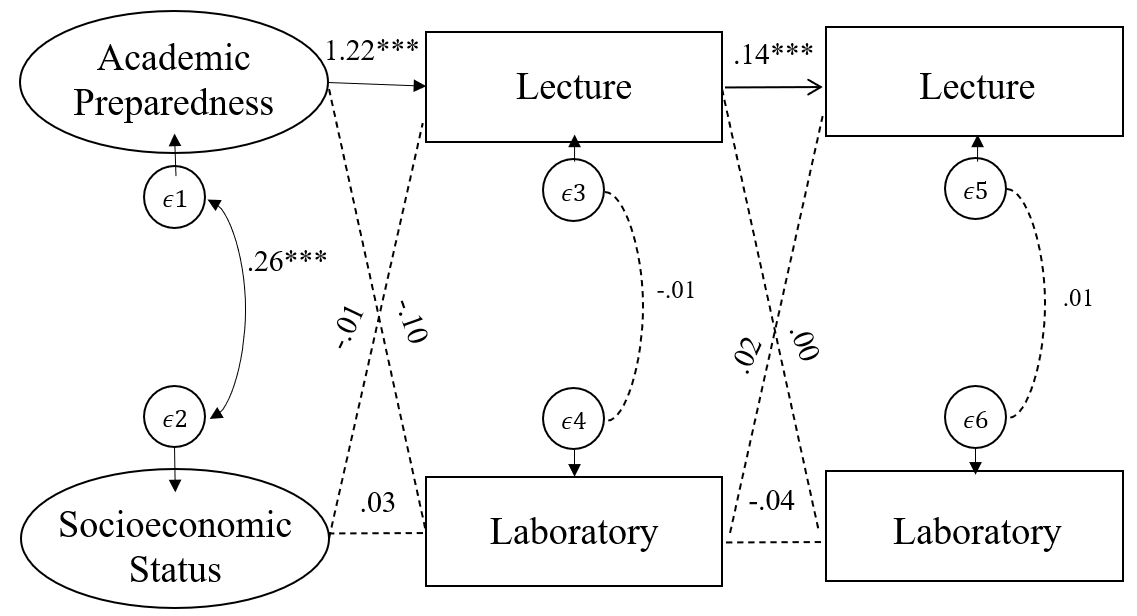

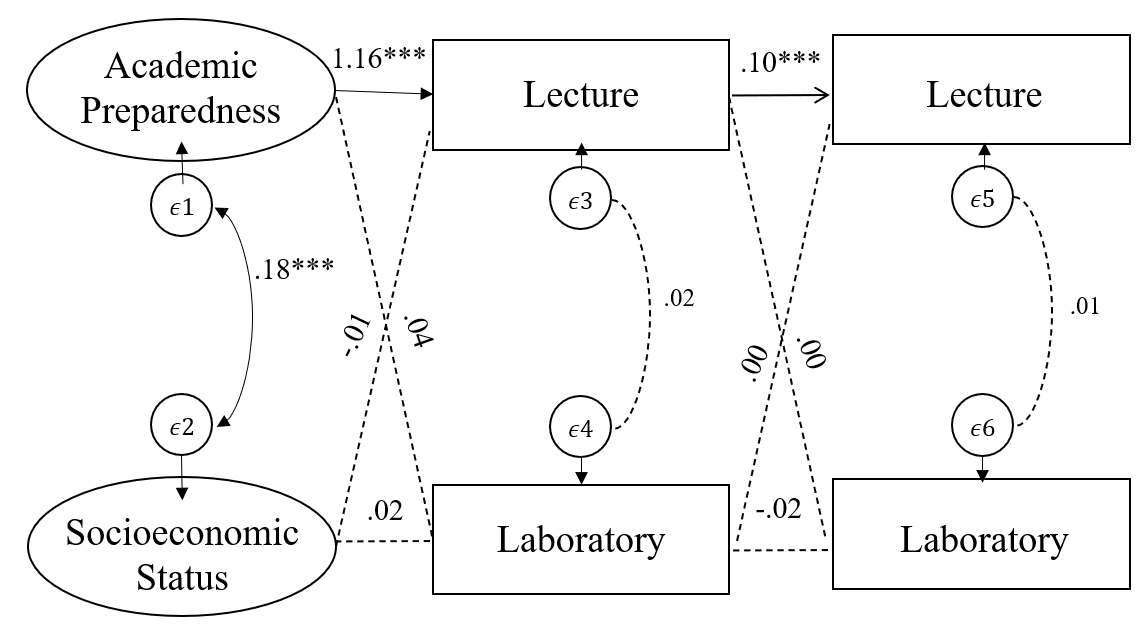


**Figure A4.** Multi-group structural equation model representing the associations between lecture and inquiry-based lab performance in physics course series including transfer and international students; left: LM students, right: non-LM students; latent covariates for academic preparedness and SES are included but not shown; dashed lines describe non-significant path estimate; *p < .05., **p < .01., ***p < .001.


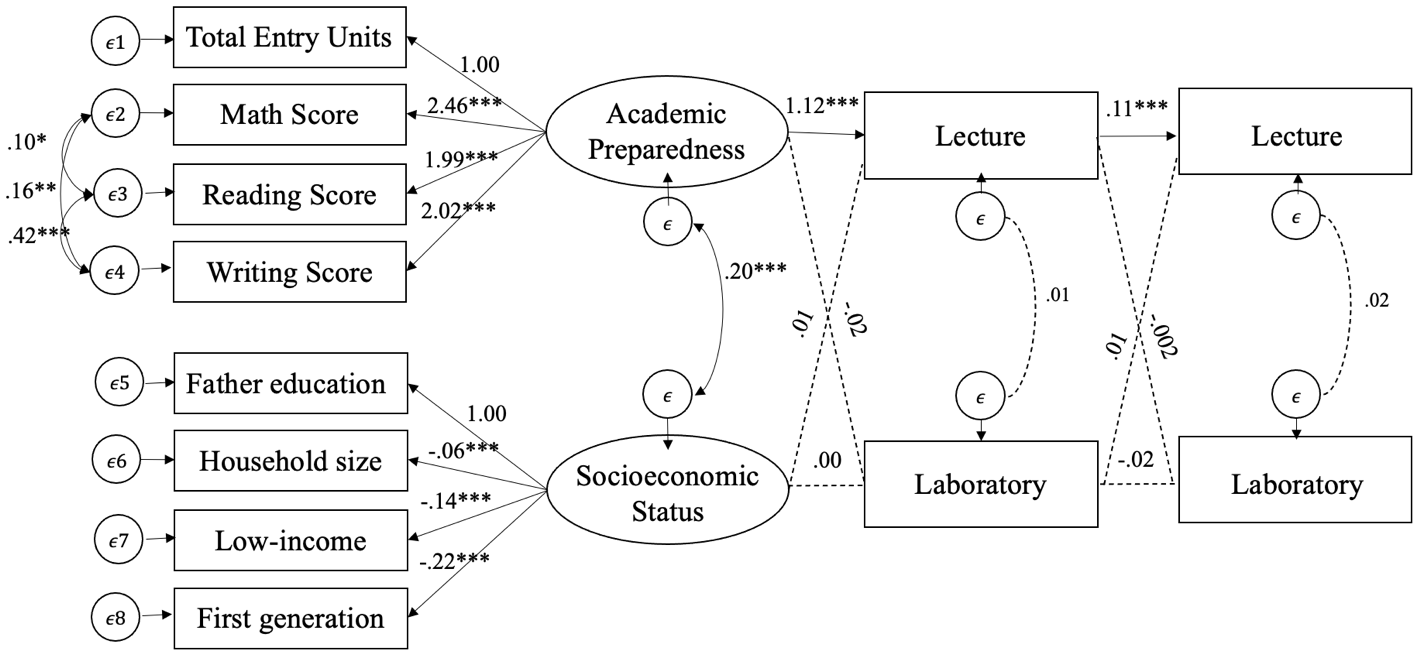


**Figure A5.** Robustness check, cross-lagged panel analysis for Physics (RQ2) using mother’s education level (instead of father’s education level).

**Table A1.** Demographic information of study sample by language group including transfer and international students.

|  | Combined | | LM students | | Non-LM students | |
| --- | --- | --- | --- | --- | --- | --- |
|  | N | % | N | % | N | % |
| Physics |  |  |  |  |  |  |
| Home language |  |  |  |  |  |  |
| English only | 1,619 | 34 |  |  | 1,619 | 52 |
| English and another language | 1,509 | 32 |  |  | 1,509 | 48 |
| Another language | 1,613 | 34 | 1,613 | 100 |  |  |
| Race/Ethnicity |  |  |  |  |  |  |
| White | 849 | 19 | 106 | 7 | 743 | 25 |
| Black/African American | 68 | 1 | 5 | .3 | 63 | 2 |
| Latino/Hispanic | 868 | 19 | 329 | 21 | 539 | 18 |
| Asian/Asian American/Pacific Islander | 2,537 | 55 | 1,056 | 68 | 1,481 | 49 |
| Other | 265 | 6 | 68 | 4 | 197 | 7 |
| Gender |  |  |  |  |  |  |
| Male | 3,466 | 73 | 1,125 | 70 | 2,341 | 75 |
| Female | 1,255 | 27 | 482 | 30 | 773 | 25 |
| Chemistry |  |  |  |  |  |  |
| Home language |  |  |  |  |  |  |
| English only | 970 | 33 |  |  | 970 | 41 |
| English and another language | 1,383 | 48 |  |  | 1,383 | 59 |
| Another language | 558 | 19 | 558 | 100 |  |  |
| Race/Ethnicity |  |  |  |  |  |  |
| White | 432 | 15 | 56 | 10 | 376 | 17 |
| Black/African American | 74 | 3 | 8 | 1 | 66 | 3 |
| Latino/Hispanic | 368 | 13 | 103 | 19 | 265 | 12 |
| Asian/Asian American/Pacific Islander | 1,688 | 60 | 340 | 63 | 1,348 | 60 |
| Other | 235 | 8 | 33 | 6 | 202 | 9 |
| Gender |  |  |  |  |  |  |
| Male | 1,146 | 39 | 198 | 35 | 948 | 40 |
| Female | 1,756 | 61 | 360 | 65 | 1,396 | 60 |

*Notes.* LM: Language minority; percentages may not add up to 100% due to rounding.

**Table A2.** Descriptive statistics and standardized mean differences by language group including transfer and international students.

|  | LM | | Non-LM | | Effect size and 95% CI | | |
| --- | --- | --- | --- | --- | --- | --- | --- |
|  | M | SD | M | SD | d | Low CI | High CI |
| Physics |  |  |  |  |  |  |  |
| Course grade |  |  |  |  |  |  |  |
| Course 1 Lec | 2.99 | .86 | 2.86 | .81 | -.16** | -.22 | .10 |
| Course 1 Lab | 3.77 | .58 | 3.78 | .56 | .08* | -.05 | .07 |
| Course 2 Lec | 2.75 | .97 | 2.74 | .96 | -.00 | -.06 | .06 |
| Course 2 Lab | 3.00 | .65 | 2.98 | .61 | -.03 | -.09 | .04 |
| Academic preparedness |  |  |  |  |  |  |  |
| Entry units | 28.05 | 22.5 | 19.82 | 23.66 | .36*** | .30 | .42 |
| Math score | 84.47 | 9.41 | 86.22 | 9.78 | .18*** | -.24 | -.12 |
| Reading score | 69.74 | 12.55 | 75.79 | 12.59 | .48*** | .42 | .54 |
| Writing score | 72.95 | 11.32 | 74.99 | 11.94 | .17*** | .11 | .23 |
| Family background |  |  |  |  |  |  |  |
| Father's education | 4.28 | 2.07 | 5.08 | 1.79 | .42*** | .36 | .49 |
| Low-income status | 37% |  | 22% |  |  |  |  |
| First-generation status | 48% |  | 32% |  |  |  |  |
| Household size | 4.24 | 1.30 | 4.16 | 1.16 | -.07* | -.14 | .01 |
| Chemistry |  |  |  |  |  |  |  |
| Course grade |  |  |  |  |  |  |  |
| Course 1 Lec | 2.45 | 1.02 | 2.62 | .92 | .17** | .08 | .27 |
| Course 1 Lab | 3.23 | .54 | 3.23 | .58 | .01 | -.10 | .12 |
| Course 2 Lec | 2.33 | 1.07 | 2.37 | 1.04 | .04 | -.07 | .15 |
| Course 2 Lab | 3.07 | .70 | 3.03 | .67 | -.05 | -.16 | .06 |
| Academic preparedness |  |  |  |  |  |  |  |
| Entry units | 23.21 | 18.84 | 24.90 | 20.71 | .08 | -.01 | .18 |
| Math score | 74.61 | 12.69 | 74.66 | 11.41 | .004 | -.09 | .10 |
| Reading score | 63.07 | 14.34 | 67.46 | 12.78 | .33*** | .24 | .43 |
| Writing score | 65.87 | 13.51 | 68.31 | 11.88 | .20*** | .11 | .29 |
| Family background |  |  |  |  |  |  |  |
| Father's education | 4.36 | 2.07 | 5.12 | 1.79 | .42*** | .32 | .51 |
| Low-income status | 43% |  | 23% |  |  |  |  |
| First-generation status | 45% |  | 32% |  |  |  |  |
| Household size | 4.23 | 1.07 | 4.30 | 1.20 | -.0* | -.04 | .16 |

*Notes.* LM: Language minority; lower and upper bounds of the 95% Confidence Interval (CI), positive value indicate direction of effect in favor of non-LM students, whereas negative values are in favor of LM students. *p < .05, **p < .01, ***p < .001.

**Table A3.** Model fit indices comparing mothers’ educational level and fathers’ educational level for Physics (RQ2); models for Chemistry did not converge using mother’s education.

|  | Using mother’s education | Using father’s education |
| --- | --- | --- |
| df(45) χ^2^ | 119.47 (44) | 105.97 |
| p-value | < .001 | < .001 |
| CFI | .99 | .99 |
| TLI | .98 | .99 |
| RMSEA | .025 [.020, .030] | .020 [.020, .030] |
| SRMR | .019 | .02 |

**Table A4**. Raw output data for Figure 1. Cross-lagged panel model (Chemistry).

|  | Estimate | Standard error | z-value | p-value |
| --- | --- | --- | --- | --- |
| Academic preparedness |  |  |  |  |
| Total entry units | 1.000 |  |  |  |
| Mathematics score | 1.846 | 0.180 | 10.229 | <0.001 |
| Reading score | 1.443 | 0.143 | 10.056 | <0.001 |
| Writing score | 1.669 | 0.164 | 10.147 | <0.001 |
| Socioeconomic status |  |  |  |  |
| Father’s education level | 1.000 |  |  |  |
| Household size | 0.002 | 0.018 | 0.093 | 0.926 |
| Low-income status | -0.126 | 0.007 | -18.355 | <0.001 |
| First-generation status | -0.238 | 0.007 | -35.154 | <0.001 |
| Lecture 1 |  |  |  |  |
| Academic preparedness | 0.675 | 0.063 | 10.736 | <0.001 |
| Socioeconomic status | -0.022 | 0.013 | -1.645 | 0.100 |
| Lab 1 |  |  |  |  |
| Academic preparedness | -0.023 | 0.041 | -0.563 | 0.574 |
| Socioeconomic status | 0.018 | 0.011 | 1.675 | 0.094 |
| Lecture 2 |  |  |  |  |
| Lecture 1 | -0.027 | 0.034 | -0.783 | 0.434 |
| Lab 1 | 0.010 | 0.043 | 0.229 | 0.819 |
| Lab 2 |  |  |  |  |
| Lecture 1 | 0.033 | 0.022 | 1.465 | 0.143 |
| Lab 1 | 0.675 | 0.063 | 10.736 | <0.001 |
| *Covariances* |  |  |  |  |
| Academic preparedness |  |  |  |  |
| Socioeconomic status | 0.321 | 0.032 | 10.057 | <0.001 |
| Mathematics score |  |  |  |  |
| Reading score | -0.092 | 0.041 | -2.207 | 0.027 |
| Writing score | -0.130 | 0.045 | -2.888 | 0.004 |
| Reading score |  |  |  |  |
| Writing score | 0.099 | 0.042 | 2.335 | 0.020 |
| Lecture 1 |  |  |  |  |
| Lab 1 | 0.004 | 0.010 | 0.407 | 0.684 |
| Lecture 2 |  |  |  |  |
| Lab 2 | 0.021 | 0.018 | 1.171 | 0.242 |
| *Variances* |  |  |  |  |
| Total entry units | 0.814 | 0.172 | 4.742 | <0.001 |
| Mathematics score | 0.403 | 0.056 | 7.236 | <0.001 |
| Reading score | 0.581 | 0.046 | 12.587 | <0.001 |
| Writing score | 0.466 | 0.051 | 9.183 | <0.001 |
| Father’s education level | 0.487 | 0.084 | 5.822 | <0.001 |
| Household size | 1.212 | 0.068 | 17.820 | <0.001 |
| Low-income status | 0.145 | 0.005 | 27.433 | <0.001 |
| First-generation status | 0.062 | 0.005 | 12.733 | <0.001 |
| Lecture 1 | 0.465 | 0.016 | 28.767 | <0.001 |
| Lab 1 | 0.338 | 0.016 | 20.512 | <0.001 |
| Lecture 2 | 1.092 | 0.035 | 31.139 | <0.001 |
| Lab 2 | 0.458 | 0.020 | 23.113 | <0.001 |
| Academic preparedness | 0.182 | 0.024 | 7.708 | <0.001 |
| Socioeconomic status | 2.904 | 0.121 | 23.929 | <0.001 |

**Table A5**. Raw output data for Figure 2. Cross-lagged panel model (Physics).

|  | Estimate | Standard error | z-value | p-value |
| --- | --- | --- | --- | --- |
| Academic preparedness |  |  |  |  |
| Total entry units | 1.000 |  |  |  |
| Mathematics score | 2.477 | 0.230 | 10.793 | <0.001 |
| Reading score | 2.020 | 0.182 | 11.110 | <0.001 |
| Writing score | 2.087 | 0.190 | 10.997 | <0.001 |
| Socioeconomic status |  |  |  |  |
| Father’s education level | 1.000 |  |  |  |
| Household size | -0.056 | 0.014 | -4.087 | <0.001 |
| Low-income status | -0.130 | 0.005 | -25.828 | <0.001 |
| First-generation status | -0.224 | 0.006 | -40.095 | <0.001 |
| Lecture 1 |  |  |  |  |
| Academic preparedness | 1.047 | 0.088 | 11.903 | <0.001 |
| Socioeconomic status | 0.007 | 0.011 | 0.629 | 0.529 |
| Lab 1 |  |  |  |  |
| Academic preparedness | -0.010 | 0.045 | -0.223 | 0.823 |
| Socioeconomic status | 0.000 | 0.007 | 0.014 | 0.988 |
| Lecture 2 |  |  |  |  |
| Lecture 1 | 0.107 | 0.024 | 4.475 | <0.001 |
| Lab 1 | 0.010 | 0.034 | 0.293 | 0.769 |
| Lab 2 |  |  |  |  |
| Lecture 1 | 0.001 | 0.014 | 0.087 | 0.930 |
| Lab 1 | -0.025 | 0.019 | -1.343 | 0.179 |
| *Covariances* |  |  |  |  |
| Academic preparedness |  |  |  |  |
| Socioeconomic status | 0.227 | 0.023 | 9.731 | <0.001 |
| Mathematics score |  |  |  |  |
| Reading score | 0.062 | 0.041 | 1.515 | 0.130 |
| Writing score | 0.114 | 0.043 | 2.661 | 0.008 |
| Reading score |  |  |  |  |
| Writing score | 0.360 | 0.042 | 8.634 | <0.001 |
| Lecture 1 |  |  |  |  |
| Lab 1 | 0.010 | 0.008 | 1.193 | 0.233 |
| Lecture 2 |  |  |  |  |
| Lab 2 | 0.008 | 0.011 | 0.682 | 0.496 |
| *Variances* |  |  |  |  |
| Total entry units | 0.552 | 0.031 | 17.803 | <0.001 |
| Mathematics score | 0.460 | 0.052 | 8.915 | <0.001 |
| Reading score | 0.618 | 0.044 | 14.013 | <0.001 |
| Writing score | 0.665 | 0.046 | 14.580 | <0.001 |
| Father’s education level | 0.430 | 0.079 | 5.452 | <0.001 |
| Household size | 1.372 | 0.058 | 23.461 | <0.001 |
| Low-income status | 0.160 | 0.004 | 38.292 | <0.001 |
| First-generation status | 0.075 | 0.005 | 16.234 | <0.001 |
| Lecture 1 | 0.506 | 0.016 | 31.108 | <0.001 |
| Lab 1 | 0.320 | 0.025 | 13.052 | <0.001 |
| Lecture 2 | 0.935 | 0.026 | 35.539 | <0.001 |
| Lab 2 | 0.384 | 0.017 | 23.133 | <0.001 |
| Academic preparedness | 0.105 | 0.014 | 7.606 | <0.001 |
| Socioeconomic status | 3.380 | 0.107 | 31.538 | <0.001 |

**Table A6**. Raw output data for Figure 3. Multigroup structural equation model (Chemistry).

|  | Estimate | Standard error | z-value | p-value |
| --- | --- | --- | --- | --- |
| *Group: Language Minority Students* | | | | |
| Academic preparedness |  |  |  |  |
| Total entry units | 1.000 |  |  |  |
| Mathematics score | 2.875 | 0.768 | 3.745 | <0.001 |
| Reading score | 1.779 | 0.510 | 3.488 | <0.001 |
| Writing score | 2.368 | 0.622 | 3.805 | <0.001 |
| Socioeconomic status |  |  |  |  |
| Father’s education level | 1.000 |  |  |  |
| Household size | -0.017 | 0.031 | -0.550 | 0.583 |
| Low-income status | -0.147 | 0.015 | -9.684 | <0.001 |
| First-generation status | -0.230 | 0.012 | -19.319 | <0.001 |
| Lecture 1 |  |  |  |  |
| Academic preparedness | 1.041 | 0.293 | 3.554 | <0.001 |
| Socioeconomic status | -0.025 | 0.041 | -0.598 | 0.550 |
| Lab 1 |  |  |  |  |
| Academic preparedness | -0.144 | 0.117 | -1.237 | 0.216 |
| Socioeconomic status | 0.065 | 0.023 | 2.870 | 0.004 |
| Lecture 2 |  |  |  |  |
| Lecture 1 | 0.012 | 0.085 | 0.147 | 0.883 |
| Lab 1 | -0.035 | 0.105 | -0.332 | 0.740 |
| Lab 2 |  |  |  |  |
| Lecture 1 | -0.004 | 0.050 | -0.079 | 0.937 |
| Lab 1 | 0.154 | 0.080 | 1.932 | 0.053 |
| *Covariances* |  |  |  |  |
| Academic preparedness |  |  |  |  |
| Socioeconomic status | 0.330 | 0.095 | 3.484 | <0.001 |
| Mathematics score |  |  |  |  |
| Reading score | -0.149 | 0.176 | -0.846 | 0.397 |
| Writing score | -0.249 | 0.201 | -1.241 | 0.215 |
| Reading score |  |  |  |  |
| Writing score | 0.262 | 0.172 | 1.523 | 0.128 |
| Lecture 1 |  |  |  |  |
| Lab 1 | 0.023 | 0.026 | 0.906 | 0.365 |
| Lecture 2 |  |  |  |  |
| Lab 2 | 0.003 | 0.040 | 0.072 | 0.942 |
| *Intercepts* |  |  |  |  |
| Total entry units | 0.123 | 0.058 | 2.126 | 0.034 |
| Mathematics score | -0.022 | 0.065 | -0.343 | 0.732 |
| Reading score | -0.407 | 0.062 | -6.593 | <0.001 |
| Writing score | -0.270 | 0.065 | -4.165 | <0.001 |
| Father’s education level | 4.455 | 0.119 | 37.488 | <0.001 |
| Household size | 4.216 | 0.057 | 74.448 | <0.001 |
| Low-income status | 0.421 | 0.029 | 14.578 | <0.001 |
| First-generation status | 0.455 | 0.029 | 15.629 | <0.001 |
| Lecture 1 | 2.846 | 0.045 | 63.296 | <0.001 |
| Lab 1 | 3.199 | 0.032 | 98.666 | <0.001 |
| Lecture 2 | 2.442 | 0.404 | 6.052 | <0.001 |
| Lab 2 | 2.584 | 0.307 | 8.403 | <0.001 |
| *Variances* |  |  |  |  |
| Total entry units | 0.850 | 0.192 | 4.420 | <0.001 |
| Mathematics score | 0.218 | 0.222 | 0.984 | 0.325 |
| Reading score | 0.731 | 0.160 | 4.568 | <0.001 |
| Writing score | 0.548 | 0.207 | 2.646 | 0.008 |
| Father’s education level | 0.561 | 0.184 | 3.050 | 0.002 |
| Household size | 0.935 | 0.089 | 10.456 | <0.001 |
| Low-income status | 0.167 | 0.014 | 11.673 | <0.001 |
| First-generation status | 0.059 | 0.011 | 5.247 | <0.001 |
| Lecture 1 | 0.474 | 0.053 | 8.993 | <0.001 |
| Lab 1 | 0.296 | 0.026 | 11.192 | <0.001 |
| Lecture 2 | 1.139 | 0.085 | 13.469 | <0.001 |
| Lab 2 | 0.457 | 0.050 | 9.216 | <0.001 |
| Academic preparedness | 0.121 | 0.045 | 2.680 | 0.007 |
| Socioeconomic status | 3.564 | 0.267 | 13.324 | <0.001 |
| *Group: Non-Language Minority Students* | | | | |
| Academic preparedness |  |  |  |  |
| Total entry units | 1.000 |  |  |  |
| Mathematics score | 1.644 | 0.171 | 9.604 | <0.001 |
| Reading score | 1.294 | 0.134 | 9.666 | <0.001 |
| Writing score | 1.471 | 0.157 | 9.377 | <0.001 |
| Socioeconomic status |  |  |  |  |
| Father’s education level | 1.000 |  |  |  |
| Household size | 0.003 | 0.021 | 0.144 | 0.886 |
| Low-income status | -0.116 | 0.008 | -14.906 | <0.001 |
| First-generation status | -0.243 | 0.009 | -28.588 | <0.001 |
| Lecture 1 |  |  |  |  |
| Academic preparedness | 0.639 | 0.063 | 10.143 | <0.001 |
| Socioeconomic status | -0.025 | 0.014 | -1.776 | 0.076 |
| Lab 1 |  |  |  |  |
| Academic preparedness | -0.012 | 0.045 | -0.262 | 0.793 |
| Socioeconomic status | 0.006 | 0.012 | 0.502 | 0.616 |
| Lecture 2 |  |  |  |  |
| Lecture 1 | -0.035 | 0.037 | -0.943 | 0.346 |
| Lab 1 | 0.017 | 0.047 | 0.367 | 0.713 |
| Lab 2 |  |  |  |  |
| Lecture 1 | 0.040 | 0.025 | 1.611 | 0.107 |
| Lab 1 | 0.044 | 0.030 | 1.485 | 0.137 |
| *Covariances* |  |  |  |  |
| Academic preparedness |  |  |  |  |
| Socioeconomic status | 0.314 | 0.033 | 9.515 | <0.001 |
| Mathematics score |  |  |  |  |
| Reading score | -0.049 | 0.038 | -1.286 | 0.198 |
| Writing score | -0.086 | 0.042 | -2.044 | 0.041 |
| Reading score |  |  |  |  |
| Writing score | 0.097 | 0.040 | 2.450 | 0.014 |
| Lecture 1 |  |  |  |  |
| Lab 1 | 0.001 | 0.011 | 0.104 | 0.917 |
| Lecture 2 |  |  |  |  |
| Lab 2 | 0.025 | 0.020 | 1.259 | 0.208 |
| *Intercepts* |  |  |  |  |
| Total entry units | 0.067 | 0.026 | 2.541 | 0.011 |
| Mathematics score | -0.087 | 0.026 | -3.354 | 0.001 |
| Reading score | -0.092 | 0.025 | -3.658 | <0.001 |
| Writing score | -0.115 | 0.025 | -4.563 | <0.001 |
| Father’s education level | 5.138 | 0.047 | 110.267 | <0.001 |
| Household size | 4.298 | 0.029 | 145.904 | <0.001 |
| Low-income status | 0.223 | 0.011 | 20.489 | <0.001 |
| First-generation status | 0.326 | 0.012 | 26.580 | <0.001 |
| Lecture 1 | 2.836 | 0.019 | 148.72 | <0.001 |
| Lab 1 | 3.230 | 0.015 | 210.066 | <0.001 |
| Lecture 2 | 2.412 | 0.187 | 12.913 | <0.001 |
| Lab 2 | 2.773 | 0.121 | 22.867 | <0.001 |
| *Variances* |  |  |  |  |
| Total entry units | 0.798 | 0.204 | 3.921 | <0.001 |
| Mathematics score | 0.437 | 0.054 | 8.120 | <0.001 |
| Reading score | 0.573 | 0.044 | 12.911 | <0.001 |
| Writing score | 0.481 | 0.049 | 9.817 | <0.001 |
| Father’s education level | 0.491 | 0.095 | 5.187 | <0.001 |
| Household size | 1.266 | 0.079 | 15.956 | <0.001 |
| Low-income status | 0.137 | 0.006 | 24.115 | <0.001 |
| First-generation status | 0.062 | 0.006 | 10.932 | <0.001 |
| Lecture 1 | 0.456 | 0.017 | 26.786 | <0.001 |
| Lab 1 | 0.345 | 0.019 | 18.139 | <0.001 |
| Lecture 2 | 1.083 | 0.039 | 28.090 | <0.001 |
| Lab 2 | 0.458 | 0.022 | 21.206 | <0.001 |
| Academic preparedness | 0.203 | 0.028 | 7.260 | <0.001 |
| Socioeconomic status | 2.676 | 0.135 | 19.866 | <0.001 |

**Table A7**. Raw output data for Figure 4. Multigroup structural equation model (Physics).

|  | Estimate | Standard error | z-value | p-value |
| --- | --- | --- | --- | --- |
| *Group: Language Minority Students* | | | | |
| Academic preparedness |  |  |  |  |
| Total entry units | 1.000 |  |  |  |
| Mathematics score | 2.463 | 0.501 | 4.917 | <0.001 |
| Reading score | 1.586 | 0.300 | 5.285 | <0.001 |
| Writing score | 1.955 | 0.358 | 5.464 | <0.001 |
| Socioeconomic status |  |  |  |  |
| Father’s education level | 1.000 |  |  |  |
| Household size | -0.107 | 0.025 | -4.249 | <0.001 |
| Low-income status | -0.128 | 0.010 | -12.768 | <0.001 |
| First-generation status | -0.211 | 0.010 | -20.719 | <0.001 |
| Lecture 1 |  |  |  |  |
| Academic preparedness | 1.150 | 0.240 | 4.787 | <0.001 |
| Socioeconomic status | -0.008 | 0.023 | -0.365 | 0.715 |
| Lab 1 |  |  |  |  |
| Academic preparedness | -0.105 | 0.109 | -0.968 | 0.333 |
| Socioeconomic status | 0.032 | 0.015 | 2.133 | 0.033 |
| Lecture 2 |  |  |  |  |
| Lecture 1 | 0.143 | 0.048 | 3.008 | 0.003 |
| Lab 1 | 0.019 | 0.064 | 0.301 | 0.763 |
| Lab 2 |  |  |  |  |
| Lecture 1 | 0.000 | 0.031 | 0.001 | 0.999 |
| Lab 1 | -0.036 | 0.036 | -1.014 | 0.311 |
| *Covariances* |  |  |  |  |
| Academic preparedness |  |  |  |  |
| Socioeconomic status | 0.265 | 0.056 | 4.698 | <0.001 |
| Mathematics score |  |  |  |  |
| Reading score | 0.165 | 0.074 | 2.238 | 0.025 |
| Writing score | 0.145 | 0.077 | 1.871 | 0.061 |
| Reading score |  |  |  |  |
| Writing score | 0.516 | 0.085 | 6.074 | <0.001 |
| Lecture 1 |  |  |  |  |
| Lab 1 | -0.013 | 0.018 | -0.701 | 0.483 |
| Lecture 2 |  |  |  |  |
| Lab 2 | 0.007 | 0.025 | 0.279 | 0.780 |
| *Intercepts* |  |  |  |  |
| Total entry units | 0.053 | 0.032 | 1.673 | 0.094 |
| Mathematics score | -0.305 | 0.042 | -7.319 | <0.001 |
| Reading score | -0.287 | 0.040 | -7.215 | <0.001 |
| Writing score | -0.271 | 0.040 | -6.719 | <0.001 |
| Father’s education level | 3.547 | 0.077 | 45.95 | <0.001 |
| Household size | 4.350 | 0.049 | 87.985 | <0.001 |
| Low-income status | 0.582 | 0.019 | 31.003 | <0.001 |
| First-generation status | 0.658 | 0.018 | 36.5 | <0.001 |
| Lecture 1 | 2.825 | 0.030 | 92.957 | <0.001 |
| Lab 1 | 3.781 | 0.023 | 167.12 | <0.001 |
| Lecture 2 | 2.276 | 0.273 | 8.344 | <0.001 |
| Lab 2 | 3.137 | 0.162 | 19.321 | <0.001 |
| *Variances* |  |  |  |  |
| Total entry units | 0.589 | 0.099 | 5.938 | <0.001 |
| Mathematics score | 0.546 | 0.094 | 5.788 | <0.001 |
| Reading score | 0.822 | 0.088 | 9.300 | <0.001 |
| Writing score | 0.714 | 0.093 | 7.665 | <0.001 |
| Father’s education level | 0.537 | 0.159 | 3.366 | 0.001 |
| Household size | 1.648 | 0.154 | 10.737 | <0.001 |
| Low-income status | 0.185 | 0.009 | 21.31 | <0.001 |
| First-generation status | 0.066 | 0.009 | 7.217 | <0.001 |
| Lecture 1 | 0.500 | 0.037 | 13.649 | <0.001 |
| Lab 1 | 0.351 | 0.055 | 6.320 | <0.001 |
| Lecture 2 | 0.926 | 0.053 | 17.516 | <0.001 |
| Lab 2 | 0.443 | 0.039 | 11.454 | <0.001 |
| Academic preparedness | 0.108 | 0.036 | 3.019 | 0.003 |
| Socioeconomic status | 3.581 | 0.220 | 16.305 | <0.001 |
| *Group: Non-Language Minority Students* | | | | |
| Academic preparedness |  |  |  |  |
| Total entry units | 1.000 |  |  |  |
| Mathematics score | 2.606 | 0.292 | 8.918 | <0.001 |
| Reading score | 2.118 | 0.234 | 9.064 | <0.001 |
| Writing score | 2.154 | 0.244 | 8.833 | <0.001 |
| Socioeconomic status |  |  |  |  |
| Father’s education level | 1.000 |  |  |  |
| Household size | -0.026 | 0.017 | -1.503 | 0.133 |
| Low-income status | -0.111 | 0.007 | -16.792 | <0.001 |
| First-generation status | -0.235 | 0.009 | -27.602 | <0.001 |
| Lecture 1 |  |  |  |  |
| Academic preparedness | 1.073 | 0.100 | 10.742 | <0.001 |
| Socioeconomic status | 0.014 | 0.013 | 1.051 | 0.293 |
| Lab 1 |  |  |  |  |
| Academic preparedness | 0.029 | 0.052 | 0.551 | 0.581 |
| Socioeconomic status | -0.012 | 0.008 | -1.559 | 0.119 |
| Lecture 2 |  |  |  |  |
| Lecture 1 | 0.096 | 0.028 | 3.450 | 0.001 |
| Lab 1 | 0.008 | 0.041 | 0.189 | 0.850 |
| Lab 2 |  |  |  |  |
| Lecture 1 | 0.002 | 0.016 | 0.134 | 0.893 |
| Lab 1 | -0.022 | 0.022 | -0.962 | 0.336 |
| *Covariances* |  |  |  |  |
| Academic preparedness |  |  |  |  |
| Socioeconomic status | 0.176 | 0.023 | 7.637 | <0.001 |
| Mathematics score |  |  |  |  |
| Reading score | 0.044 | 0.052 | 0.850 | 0.395 |
| Writing score | 0.107 | 0.054 | 1.972 | 0.049 |
| Reading score |  |  |  |  |
| Writing score | 0.327 | 0.050 | 6.500 | <0.001 |
| Lecture 1 |  |  |  |  |
| Lab 1 | 0.018 | 0.010 | 1.842 | 0.066 |
| Lecture 2 |  |  |  |  |
| Lab 2 | 0.008 | 0.013 | 0.625 | 0.532 |
| *Intercepts* |  |  |  |  |
| Total entry units | 0.160 | 0.018 | 9.032 | <0.001 |
| Mathematics score | -0.136 | 0.023 | -5.954 | <0.001 |
| Reading score | 0.076 | 0.022 | 3.433 | 0.001 |
| Writing score | -0.032 | 0.023 | -1.384 | 0.166 |
| Father’s education level | 4.997 | 0.039 | 126.788 | <0.001 |
| Household size | 4.206 | 0.025 | 168.54 | <0.001 |
| Low-income status | 0.232 | 0.009 | 24.840 | <0.001 |
| First-generation status | 0.356 | 0.011 | 33.586 | <0.001 |
| Lecture 1 | 2.896 | 0.017 | 166.548 | <0.001 |
| Lab 1 | 3.773 | 0.012 | 307.111 | <0.001 |
| Lecture 2 | 2.429 | 0.170 | 14.291 | <0.001 |
| Lab 2 | 3.054 | 0.096 | 31.715 | <0.001 |
| *Variances* |  |  |  |  |
| Total entry units | 0.546 | 0.024 | 22.56 | <0.001 |
| Mathematics score | 0.415 | 0.065 | 6.332 | <0.001 |
| Reading score | 0.568 | 0.053 | 10.766 | <0.001 |
| Writing score | 0.662 | 0.055 | 12.022 | <0.001 |
| Father’s education level | 0.416 | 0.095 | 4.385 | <0.001 |
| Household size | 1.272 | 0.057 | 22.139 | <0.001 |
| Low-income status | 0.144 | 0.005 | 29.879 | <0.001 |
| First-generation status | 0.077 | 0.006 | 13.087 | <0.001 |
| Lecture 1 | 0.503 | 0.019 | 27.168 | <0.001 |
| Lab 1 | 0.308 | 0.027 | 11.54 | <0.001 |
| Lecture 2 | 0.937 | 0.030 | 30.926 | <0.001 |
| Lab 2 | 0.365 | 0.018 | 20.258 | <0.001 |
| Academic preparedness | 0.095 | 0.015 | 6.545 | <0.001 |
| Socioeconomic status | 2.760 | 0.125 | 22.018 | <0.001 |

**Table A8**. Raw output data for Figure A1. Cross-lagged panel model (Chemistry; w/ transfer and int. students).

|  | Estimate | Standard error | z-value | p-value |
| --- | --- | --- | --- | --- |
| Academic preparedness |  |  |  |  |
| Total entry units | 1.000 |  |  |  |
| Mathematics score | 1.835 | 0.184 | 9.995 | <0.001 |
| Reading score | 1.406 | 0.145 | 9.692 | <0.001 |
| Writing score | 1.646 | 0.166 | 9.918 | <0.001 |
| Socioeconomic status |  |  |  |  |
| Father’s education level | 1.000 |  |  |  |
| Household size | 0.002 | 0.018 | 0.114 | 0.909 |
| Low-income status | -0.126 | 0.007 | -18.303 | <0.001 |
| First-generation status | -0.238 | 0.007 | -34.977 | <0.001 |
| Lecture 1 |  |  |  |  |
| Academic preparedness | 0.709 | 0.071 | 9.995 | <0.001 |
| Socioeconomic status | -0.025 | 0.014 | -1.813 | 0.070 |
| Lab 1 |  |  |  |  |
| Academic preparedness | -0.030 | 0.044 | -0.696 | 0.487 |
| Socioeconomic status | 0.018 | 0.011 | 1.677 | 0.094 |
| Lecture 2 |  |  |  |  |
| Lecture 1 | -0.024 | 0.034 | -0.723 | 0.470 |
| Lab 1 | 0.004 | 0.043 | 0.105 | 0.917 |
| Lab 2 |  |  |  |  |
| Lecture 1 | 0.033 | 0.022 | 1.465 | 0.143 |
| Lab 1 | 0.063 | 0.028 | 2.240 | 0.025 |
| *Covariances* |  |  |  |  |
| Academic preparedness |  |  |  |  |
| Socioeconomic status | 0.323 | 0.032 | 10.008 | <0.001 |
| Mathematics score |  |  |  |  |
| Reading score | -0.061 | 0.045 | -1.377 | 0.168 |
| Writing score | -0.100 | 0.048 | -2.104 | 0.035 |
| Reading score |  |  |  |  |
| Writing score | 0.138 | 0.045 | 3.086 | 0.002 |
| Lecture 1 |  |  |  |  |
| Lab 1 | 0.005 | 0.010 | 0.481 | 0.630 |
| Lecture 2 |  |  |  |  |
| Lab 2 | 0.020 | 0.018 | 1.121 | 0.262 |
| *Variances* |  |  |  |  |
| Total entry units | 0.856 | 0.172 | 4.989 | <0.001 |
| Mathematics score | 0.436 | 0.057 | 7.622 | <0.001 |
| Reading score | 0.619 | 0.048 | 12.913 | <0.001 |
| Writing score | 0.504 | 0.053 | 9.594 | <0.001 |
| Father’s education level | 0.483 | 0.084 | 5.761 | <0.001 |
| Household size | 1.216 | 0.068 | 17.873 | <0.001 |
| Low-income status | 0.145 | 0.005 | 27.51 | <0.001 |
| First-generation status | 0.063 | 0.005 | 12.821 | <0.001 |
| Lecture 1 | 0.462 | 0.017 | 27.521 | <0.001 |
| Lab 1 | 0.338 | 0.016 | 20.608 | <0.001 |
| Lecture 2 | 1.091 | 0.035 | 31.176 | <0.001 |
| Lab 2 | 0.460 | 0.020 | 23.238 | <0.001 |
| Academic preparedness | 0.176 | 0.024 | 7.455 | <0.001 |
| Socioeconomic status | 2.898 | 0.121 | 23.919 | <0.001 |

**Table A9**. Raw output data for Figure A2. Cross-lagged panel model (Physics; w/ transfer and int. students).

|  | Estimate | Standard error | z-value | p-value |
| --- | --- | --- | --- | --- |
| Academic preparedness |  |  |  |  |
| Total entry units | 1.000 |  |  |  |
| Mathematics score | 2.433 | 0.225 | 10.799 | <0.001 |
| Reading score | 1.935 | 0.175 | 11.072 | <0.001 |
| Writing score | 1.997 | 0.184 | 10.876 | <0.001 |
| Socioeconomic status |  |  |  |  |
| Father’s education level | 1.000 |  |  |  |
| Household size | -0.054 | 0.014 | -3.999 | <0.001 |
| Low-income status | -0.130 | 0.005 | -25.854 | <0.001 |
| First-generation status | -0.224 | 0.006 | -40.245 | <0.001 |
| Lecture 1 |  |  |  |  |
| Academic preparedness | 1.113 | 0.102 | 10.93 | <0.001 |
| Socioeconomic status | -0.001 | 0.011 | -0.058 | 0.954 |
| Lab 1 |  |  |  |  |
| Academic preparedness | 0.000 | 0.048 | -0.003 | 0.998 |
| Socioeconomic status | 0.000 | 0.007 | -0.057 | 0.955 |
| Lecture 2 |  |  |  |  |
| Lecture 1 | 0.107 | 0.024 | 4.514 | <0.001 |
| Lab 1 | 0.006 | 0.034 | 0.182 | 0.856 |
| Lab 2 |  |  |  |  |
| Lecture 1 | 0.002 | 0.014 | 0.136 | 0.892 |
| Lab 1 | -0.027 | 0.019 | -1.438 | 0.150 |
| *Covariances* |  |  |  |  |
| Academic preparedness |  |  |  |  |
| Socioeconomic status | 0.231 | 0.024 | 9.801 | <0.001 |
| Mathematics score |  |  |  |  |
| Reading score | 0.104 | 0.043 | 2.439 | 0.015 |
| Writing score | 0.159 | 0.044 | 3.610 | <0.001 |
| Reading score |  |  |  |  |
| Writing score | 0.411 | 0.043 | 9.605 | <0.001 |
| Lecture 1 |  |  |  |  |
| Lab 1 | 0.009 | 0.008 | 1.065 | 0.287 |
| Lecture 2 |  |  |  |  |
| Lab 2 | 0.007 | 0.011 | 0.630 | 0.528 |
| *Variances* |  |  |  |  |
| Total entry units | 0.599 | 0.034 | 17.693 | <0.001 |
| Mathematics score | 0.500 | 0.052 | 9.657 | <0.001 |
| Reading score | 0.670 | 0.046 | 14.633 | <0.001 |
| Writing score | 0.720 | 0.047 | 15.452 | <0.001 |
| Father’s education level | 0.424 | 0.079 | 5.384 | <0.001 |
| Household size | 1.375 | 0.058 | 23.668 | <0.001 |
| Low-income status | 0.161 | 0.004 | 38.595 | <0.001 |
| First-generation status | 0.075 | 0.005 | 16.185 | <0.001 |
| Lecture 1 | 0.500 | 0.017 | 28.897 | <0.001 |
| Lab 1 | 0.324 | 0.025 | 13.187 | <0.001 |
| Lecture 2 | 0.931 | 0.026 | 35.704 | <0.001 |
| Lab 2 | 0.386 | 0.017 | 23.072 | <0.001 |
| Academic preparedness | 0.102 | 0.014 | 7.422 | <0.001 |
| Socioeconomic status | 3.390 | 0.107 | 31.687 | <0.001 |

**Table A10**. Raw output data for Figure A3. Multigroup structural equation model (Chemistry w/ transfer and int. students).

|  | Estimate | Standard error | z-value | p-value |
| --- | --- | --- | --- | --- |
| *Group: Language Minority Students* | | | | |
| Academic preparedness |  |  |  |  |
| Total entry units | 1.000 |  |  |  |
| Mathematics score | 3.102 | 0.894 | 3.471 | 0.001 |
| Reading score | 1.873 | 0.575 | 3.257 | 0.001 |
| Writing score | 2.497 | 0.706 | 3.539 | <0.001 |
| Socioeconomic status |  |  |  |  |
| Father’s education level | 1.000 |  |  |  |
| Household size | -0.016 | 0.030 | -0.513 | 0.608 |
| Low-income status | -0.146 | 0.015 | -9.694 | <0.001 |
| First-generation status | -0.230 | 0.012 | -19.457 | <0.001 |
| Lecture 1 |  |  |  |  |
| Academic preparedness | 1.068 | 0.319 | 3.346 | 0.001 |
| Socioeconomic status | -0.019 | 0.042 | -0.441 | 0.659 |
| Lab 1 |  |  |  |  |
| Academic preparedness | -0.145 | 0.120 | -1.208 | 0.227 |
| Socioeconomic status | 0.063 | 0.022 | 2.815 | 0.005 |
| Lecture 2 |  |  |  |  |
| Lecture 1 | 0.017 | 0.084 | 0.201 | 0.841 |
| Lab 1 | -0.059 | 0.105 | -0.559 | 0.576 |
| Lab 2 |  |  |  |  |
| Lecture 1 | -0.016 | 0.051 | -0.319 | 0.750 |
| Lab 1 | 0.178 | 0.080 | 2.218 | 0.027 |
| *Covariances* |  |  |  |  |
| Academic preparedness |  |  |  |  |
| Socioeconomic status | 0.307 | 0.094 | 3.270 | 0.001 |
| Mathematics score |  |  |  |  |
| Reading score | -0.178 | 0.196 | -0.909 | 0.363 |
| Writing score | -0.281 | 0.225 | -1.250 | 0.211 |
| Reading score |  |  |  |  |
| Writing score | 0.275 | 0.189 | 1.454 | 0.146 |
| Lecture 1 |  |  |  |  |
| Lab 1 | 0.018 | 0.026 | 0.710 | 0.478 |
| Lecture 2 |  |  |  |  |
| Lab 2 | -0.005 | 0.040 | -0.121 | 0.903 |
| *Intercepts* |  |  |  |  |
| Total entry units | 0.111 | 0.057 | 1.932 | 0.053 |
| Mathematics score | -0.010 | 0.064 | -0.151 | 0.880 |
| Reading score | -0.419 | 0.062 | -6.797 | <0.001 |
| Writing score | -0.280 | 0.065 | -4.324 | <0.001 |
| Father’s education level | 4.475 | 0.118 | 37.860 | <0.001 |
| Household size | 4.217 | 0.056 | 75.137 | <0.001 |
| Low-income status | 0.420 | 0.029 | 14.626 | <0.001 |
| First-generation status | 0.451 | 0.029 | 15.562 | <0.001 |
| Lecture 1 | 2.850 | 0.045 | 63.781 | <0.001 |
| Lab 1 | 3.199 | 0.032 | 98.878 | <0.001 |
| Lecture 2 | 2.498 | 0.404 | 6.182 | <0.001 |
| Lab 2 | 2.540 | 0.307 | 8.280 | <0.001 |
| *Variances* |  |  |  |  |
| Total entry units | 0.866 | 0.192 | 4.499 | <0.001 |
| Mathematics score | 0.177 | 0.249 | 0.710 | 0.477 |
| Reading score | 0.740 | 0.173 | 4.290 | <0.001 |
| Writing score | 0.560 | 0.228 | 2.456 | 0.014 |
| Father’s education level | 0.554 | 0.182 | 3.043 | 0.002 |
| Household size | 0.928 | 0.089 | 10.476 | <0.001 |
| Low-income status | 0.168 | 0.014 | 11.806 | <0.001 |
| First-generation status | 0.058 | 0.011 | 5.253 | <0.001 |
| Lecture 1 | 0.476 | 0.053 | 8.969 | <0.001 |
| Lab 1 | 0.298 | 0.026 | 11.332 | <0.001 |
| Lecture 2 | 1.146 | 0.085 | 13.523 | <0.001 |
| Lab 2 | 0.464 | 0.049 | 9.370 | <0.001 |
| Academic preparedness | 0.108 | 0.044 | 2.485 | 0.013 |
| Socioeconomic status | 3.567 | 0.266 | 13.404 | <0.001 |
| *Group: Non-Language Minority Students* | | | | |
| Academic preparedness |  |  |  |  |
| Total entry units | 1.000 |  |  |  |
| Mathematics score | 1.616 | 0.172 | 9.387 | <0.001 |
| Reading score | 1.244 | 0.135 | 9.221 | <0.001 |
| Writing score | 1.438 | 0.158 | 9.113 | <0.001 |
| Socioeconomic status |  |  |  |  |
| Father’s education level | 1.000 |  |  |  |
| Household size | 0.003 | 0.021 | 0.155 | 0.877 |
| Low-income status | -0.116 | 0.008 | -14.889 | <0.001 |
| First-generation status | -0.243 | 0.009 | -28.410 | <0.001 |
| Lecture 1 |  |  |  |  |
| Academic preparedness | 0.638 | 0.072 | 9.371 | <0.001 |
| Socioeconomic status | -0.031 | 0.015 | -2.037 | 0.042 |
| Lab 1 |  |  |  |  |
| Academic preparedness | -0.019 | 0.048 | -0.388 | 0.698 |
| Socioeconomic status | 0.007 | 0.013 | 0.519 | 0.604 |
| Lecture 2 |  |  |  |  |
| Lecture 1 | -0.033 | 0.037 | -0.901 | 0.368 |
| Lab 1 | 0.015 | 0.047 | 0.328 | 0.743 |
| Lab 2 |  |  |  |  |
| Lecture 1 | 0.043 | 0.025 | 1.730 | 0.084 |
| Lab 1 | 0.044 | 0.030 | 1.467 | 0.142 |
| *Covariances* |  |  |  |  |
| Academic preparedness |  |  |  |  |
| Socioeconomic status | 0.319 | 0.033 | 9.551 | <0.001 |
| Mathematics score |  |  |  |  |
| Reading score | -0.010 | 0.042 | -0.241 | 0.810 |
| Writing score | -0.049 | 0.045 | -1.099 | 0.272 |
| Reading score |  |  |  |  |
| Writing score | 0.137 | 0.042 | 3.255 | 0.001 |
| Lecture 1 |  |  |  |  |
| Lab 1 | 0.003 | 0.011 | 0.249 | 0.804 |
| Lecture 2 |  |  |  |  |
| Lab 2 | 0.026 | 0.020 | 1.305 | 0.192 |
| *Intercepts* |  |  |  |  |
| Total entry units | 0.080 | 0.027 | 2.988 | 0.003 |
| Mathematics score | -0.089 | 0.026 | -3.418 | 0.001 |
| Reading score | -0.096 | 0.025 | -3.843 | <0.001 |
| Writing score | -0.119 | 0.025 | -4.752 | <0.001 |
| Father’s education level | 5.136 | 0.046 | 110.655 | <0.001 |
| Household size | 4.299 | 0.029 | 145.923 | <0.001 |
| Low-income status | 0.224 | 0.011 | 20.556 | <0.001 |
| First-generation status | 0.325 | 0.012 | 26.591 | <0.001 |
| Lecture 1 | 2.839 | 0.019 | 149.031 | <0.001 |
| Lab 1 | 3.231 | 0.015 | 210.655 | <0.001 |
| Lecture 2 | 2.413 | 0.186 | 12.986 | <0.001 |
| Lab 2 | 2.769 | 0.121 | 22.886 | <0.001 |
| *Variances* |  |  |  |  |
| Total entry units | 0.846 | 0.204 | 4.159 | <0.001 |
| Mathematics score | 0.476 | 0.055 | 8.580 | <0.001 |
| Reading score | 0.614 | 0.046 | 13.219 | <0.001 |
| Writing score | 0.519 | 0.051 | 10.253 | <0.001 |
| Father’s education level | 0.488 | 0.095 | 5.145 | <0.001 |
| Household size | 1.272 | 0.079 | 16.016 | <0.001 |
| Low-income status | 0.137 | 0.006 | 24.168 | <0.001 |
| First-generation status | 0.062 | 0.006 | 11.018 | <0.001 |
| Lecture 1 | 0.452 | 0.018 | 25.264 | <0.001 |
| Lab 1 | 0.345 | 0.019 | 18.189 | <0.001 |
| Lecture 2 | 1.079 | 0.038 | 28.106 | <0.001 |
| Lab 2 | 0.458 | 0.022 | 21.273 | <0.001 |
| Academic preparedness | 0.197 | 0.028 | 6.992 | <0.001 |
| Socioeconomic status | 2.671 | 0.134 | 19.860 | <0.001 |

**Table A11**. Raw output data for Figure A4. Multigroup structural equation model (Physics w/transfer and int. students).

|  | Estimate | Standard error | z-value | p-value |
| --- | --- | --- | --- | --- |
| *Group: Language Minority Students* | | | | |
| Academic preparedness |  |  |  |  |
| Total entry units | 1.000 |  |  |  |
| Mathematics score | 2.559 | 0.538 | 4.752 | <0.001 |
| Reading score | 1.554 | 0.304 | 5.114 | <0.001 |
| Writing score | 1.929 | 0.364 | 5.294 | <0.001 |
| Socioeconomic status |  |  |  |  |
| Father’s education level | 1.000 |  |  |  |
| Household size | -0.108 | 0.025 | -4.348 | <0.001 |
| Low-income status | -0.129 | 0.010 | -13.175 | <0.001 |
| First-generation status | -0.211 | 0.010 | -21.428 | <0.001 |
| Lecture 1 |  |  |  |  |
| Academic preparedness | 1.217 | 0.272 | 4.471 | <0.001 |
| Socioeconomic status | -0.011 | 0.024 | -0.448 | 0.654 |
| Lab 1 |  |  |  |  |
| Academic preparedness | -0.096 | 0.114 | -0.841 | 0.400 |
| Socioeconomic status | 0.030 | 0.015 | 2.033 | 0.042 |
| Lecture 2 |  |  |  |  |
| Lecture 1 | 0.139 | 0.047 | 2.976 | 0.003 |
| Lab 1 | 0.024 | 0.064 | 0.377 | 0.706 |
| Lab 2 |  |  |  |  |
| Lecture 1 | 0.003 | 0.030 | 0.106 | 0.916 |
| Lab 1 | -0.039 | 0.036 | -1.099 | 0.272 |
| *Covariances* |  |  |  |  |
| Academic preparedness |  |  |  |  |
| Socioeconomic status | 0.258 | 0.055 | 4.704 | <0.001 |
| Mathematics score |  |  |  |  |
| Reading score | 0.178 | 0.079 | 2.256 | 0.024 |
| Writing score | 0.163 | 0.082 | 1.980 | 0.048 |
| Reading score |  |  |  |  |
| Writing score | 0.555 | 0.089 | 6.235 | <0.001 |
| Lecture 1 |  |  |  |  |
| Lab 1 | -0.011 | 0.018 | -0.611 | 0.541 |
| Lecture 2 |  |  |  |  |
| Lab 2 | 0.007 | 0.025 | 0.274 | 0.784 |
| *Intercepts* |  |  |  |  |
| Total entry units | 0.054 | 0.032 | 1.697 | 0.090 |
| Mathematics score | -0.289 | 0.041 | -7.015 | <0.001 |
| Reading score | -0.297 | 0.039 | -7.526 | <0.001 |
| Writing score | -0.277 | 0.040 | -6.894 | <0.001 |
| Father’s education level | 3.585 | 0.077 | 46.525 | <0.001 |
| Household size | 4.336 | 0.049 | 88.606 | <0.001 |
| Low-income status | 0.578 | 0.019 | 31.090 | <0.001 |
| First-generation status | 0.650 | 0.018 | 36.221 | <0.001 |
| Lecture 1 | 2.837 | 0.030 | 93.866 | <0.001 |
| Lab 1 | 3.781 | 0.022 | 169.932 | <0.001 |
| Lecture 2 | 2.264 | 0.270 | 8.388 | <0.001 |
| Lab 2 | 3.140 | 0.160 | 19.653 | <0.001 |
| *Variances* |  |  |  |  |
| Total entry units | 0.618 | 0.102 | 6.085 | <0.001 |
| Mathematics score | 0.542 | 0.099 | 5.461 | <0.001 |
| Reading score | 0.857 | 0.091 | 9.401 | <0.001 |
| Writing score | 0.770 | 0.098 | 7.829 | <0.001 |
| Father’s education level | 0.533 | 0.157 | 3.396 | 0.001 |
| Household size | 1.648 | 0.151 | 10.901 | <0.001 |
| Low-income status | 0.183 | 0.009 | 21.257 | <0.001 |
| First-generation status | 0.065 | 0.009 | 7.170 | <0.001 |
| Lecture 1 | 0.503 | 0.039 | 12.911 | <0.001 |
| Lab 1 | 0.347 | 0.054 | 6.383 | <0.001 |
| Lecture 2 | 0.920 | 0.052 | 17.779 | <0.001 |
| Lab 2 | 0.437 | 0.038 | 11.551 | <0.001 |
| Academic preparedness | 0.100 | 0.035 | 2.883 | 0.004 |
| Socioeconomic status | 3.659 | 0.217 | 16.879 | <0.001 |
| *Group: Non-Language Minority Students* | | | | |
| Academic preparedness |  |  |  |  |
| Total entry units | 1.000 |  |  |  |
| Mathematics score | 2.552 | 0.285 | 8.968 | <0.001 |
| Reading score | 2.025 | 0.224 | 9.044 | <0.001 |
| Writing score | 2.058 | 0.235 | 8.752 | <0.001 |
| Socioeconomic status |  |  |  |  |
| Father’s education level | 1.000 |  |  |  |
| Household size | -0.024 | 0.017 | -1.384 | 0.166 |
| Low-income status | -0.110 | 0.007 | -16.705 | <0.001 |
| First-generation status | -0.235 | 0.008 | -27.641 | <0.001 |
| Lecture 1 |  |  |  |  |
| Academic preparedness | 1.157 | 0.120 | 9.668 | <0.001 |
| Socioeconomic status | 0.005 | 0.014 | 0.338 | 0.736 |
| Lab 1 |  |  |  |  |
| Academic preparedness | 0.044 | 0.057 | 0.778 | 0.437 |
| Socioeconomic status | -0.013 | 0.008 | -1.557 | 0.120 |
| Lecture 2 |  |  |  |  |
| Lecture 1 | 0.097 | 0.028 | 3.506 | <0.001 |
| Lab 1 | 0.000 | 0.040 | 0.008 | 0.994 |
| Lab 2 |  |  |  |  |
| Lecture 1 | 0.002 | 0.016 | 0.117 | 0.907 |
| Lab 1 | -0.022 | 0.022 | -1.021 | 0.307 |
| *Covariances* |  |  |  |  |
| Academic preparedness |  |  |  |  |
| Socioeconomic status | 0.182 | 0.024 | 7.712 | <0.001 |
| Mathematics score |  |  |  |  |
| Reading score | 0.099 | 0.053 | 1.855 | 0.064 |
| Writing score | 0.162 | 0.055 | 2.956 | 0.003 |
| Reading score |  |  |  |  |
| Writing score | 0.383 | 0.051 | 7.555 | <0.001 |
| Lecture 1 |  |  |  |  |
| Lab 1 | 0.015 | 0.010 | 1.567 | 0.117 |
| Lecture 2 |  |  |  |  |
| Lab 2 | 0.007 | 0.013 | 0.571 | 0.568 |
| *Intercepts* |  |  |  |  |
| Total entry units | 0.177 | 0.018 | 9.668 | <0.001 |
| Mathematics score | -0.137 | 0.023 | -6.037 | <0.001 |
| Reading score | 0.072 | 0.022 | 3.288 | 0.001 |
| Writing score | -0.036 | 0.023 | -1.533 | 0.125 |
| Father’s education level | 5.002 | 0.039 | 127.473 | <0.001 |
| Household size | 4.205 | 0.025 | 168.983 | <0.001 |
| Low-income status | 0.233 | 0.009 | 24.982 | <0.001 |
| First-generation status | 0.354 | 0.011 | 33.626 | <0.001 |
| Lecture 1 | 2.898 | 0.017 | 167.299 | <0.001 |
| Lab 1 | 3.771 | 0.012 | 304.504 | <0.001 |
| Lecture 2 | 2.458 | 0.168 | 14.649 | <0.001 |
| Lab 2 | 3.057 | 0.095 | 32.312 | <0.001 |
| *Variances* |  |  |  |  |
| Total entry units | 0.599 | 0.029 | 20.459 | <0.001 |
| Mathematics score | 0.468 | 0.065 | 7.199 | <0.001 |
| Reading score | 0.624 | 0.054 | 11.580 | <0.001 |
| Writing score | 0.717 | 0.055 | 13.023 | <0.001 |
| Father’s education level | 0.410 | 0.095 | 4.326 | <0.001 |
| Household size | 1.274 | 0.057 | 22.297 | <0.001 |
| Low-income status | 0.145 | 0.005 | 30.136 | <0.001 |
| First-generation status | 0.077 | 0.006 | 13.075 | <0.001 |
| Lecture 1 | 0.493 | 0.020 | 24.874 | <0.001 |
| Lab 1 | 0.315 | 0.027 | 11.642 | <0.001 |
| Lecture 2 | 0.935 | 0.030 | 30.964 | <0.001 |
| Lab 2 | 0.368 | 0.018 | 20.078 | <0.001 |
| Academic preparedness | 0.092 | 0.014 | 6.333 | <0.001 |
| Socioeconomic status | 2.761 | 0.125 | 22.076 | <0.001 |

**Table A12**. Raw output data for Figure A5. Cross-lagged panel model (Physics; using mother’s education level instead of father’s education level).

|  | Estimate | Standard error | z-value | p-value |
| --- | --- | --- | --- | --- |
| Academic preparedness |  |  |  |  |
| Total entry units | 1.000 |  |  |  |
| Mathematics score | 2.455 | 0.234 | 10.489 | <0.001 |
| Reading score | 1.988 | 0.183 | 10.853 | <0.001 |
| Writing score | 2.024 | 0.19 | 10.664 | <0.001 |
| Socioeconomic status |  |  |  |  |
| Mother’s education level | 1.000 |  |  |  |
| Household size | -0.062 | 0.015 | -4.158 | <0.001 |
| Low-income status | -0.141 | 0.005 | -26.155 | <0.001 |
| First-generation status | -0.223 | 0.007 | -33.304 | <0.001 |
| Lecture 1 |  |  |  |  |
| Academic preparedness | 1.121 | 0.101 | 11.063 | <0.001 |
| Socioeconomic status | -0.003 | 0.011 | -0.277 | 0.782 |
| Lab 1 |  |  |  |  |
| Academic preparedness | -0.022 | 0.047 | -0.456 | 0.648 |
| Socioeconomic status | 0.010 | 0.007 | 1.338 | 0.181 |
| Lecture 2 |  |  |  |  |
| Lecture 1 | 0.109 | 0.023 | 4.636 | <0.001 |
| Lab 1 | 0.013 | 0.033 | 0.378 | 0.705 |
| Lab 2 |  |  |  |  |
| Lecture 1 | -0.002 | 0.014 | -0.127 | 0.899 |
| Lab 1 | -0.022 | 0.018 | -1.205 | 0.228 |
| *Covariances* |  |  |  |  |
| Academic preparedness |  |  |  |  |
| Socioeconomic status | 0.203 | 0.021 | 9.570 | <0.001 |
| Mathematics score |  |  |  |  |
| Reading score | 0.103 | 0.043 | 2.392 | 0.017 |
| Writing score | 0.157 | 0.044 | 3.563 | <0.001 |
| Reading score |  |  |  |  |
| Writing score | 0.417 | 0.043 | 9.642 | <0.001 |
| Lecture 1 |  |  |  |  |
| Lab 1 | 0.006 | 0.008 | 0.690 | 0.490 |
| Lecture 2 |  |  |  |  |
| Lab 2 | 0.002 | 0.011 | 0.204 | 0.838 |
| *Variances* |  |  |  |  |
| Total entry units | 0.598 | 0.033 | 17.844 | <0.001 |
| Mathematics score | 0.492 | 0.052 | 9.445 | <0.001 |
| Reading score | 0.678 | 0.047 | 14.567 | <0.001 |
| Writing score | 0.728 | 0.047 | 15.471 | <0.001 |
| Mother’s education level | 0.561 | 0.084 | 6.683 | <0.001 |
| Household size | 1.398 | 0.057 | 24.438 | <0.001 |
| Low-income status | 0.160 | 0.004 | 38.451 | <0.001 |
| First-generation status | 0.093 | 0.005 | 17.792 | <0.001 |
| Lecture 1 | 0.501 | 0.017 | 28.777 | <0.001 |
| Lab 1 | 0.325 | 0.024 | 13.269 | <0.001 |
| Lecture 2 | 0.930 | 0.026 | 35.954 | <0.001 |
| Lab 2 | 0.385 | 0.017 | 22.902 | <0.001 |
| Academic preparedness | 0.099 | 0.014 | 7.283 | <0.001 |
| Socioeconomic status | 3.035 | 0.107 | 28.294 | <0.001 |
